# Generative language models exhibit social identity biases

**DOI:** 10.1038/s43588-024-00741-1

**Published:** 2024-12-12

**Authors:** Tiancheng Hu, Yara Kyrychenko, Steve Rathje, Nigel Collier, Sander van der Linden, Jon Roozenbeek

**Affiliations:** 1 Department of Theoretical and Applied Linguistics, University of Cambridge, Cambridge, UK; 2 Department of Psychology, University of Cambridge, Cambridge, UK; 3 Department of Psychology, New York University, New York, NY USA; 4 Department of War Studies, King’s College London, London, UK

**Keywords:** Computer science, Psychology

## Abstract

Social identity biases, particularly the tendency to favor one’s own group (ingroup solidarity) and derogate other groups (outgroup hostility), are deeply rooted in human psychology and social behavior. However, it is unknown if such biases are also present in artificial intelligence systems. Here we show that large language models (LLMs) exhibit patterns of social identity bias, similarly to humans. By administering sentence completion prompts to 77 different LLMs (for instance, ‘We are…’), we demonstrate that nearly all base models and some instruction-tuned and preference-tuned models display clear ingroup favoritism and outgroup derogation. These biases manifest both in controlled experimental settings and in naturalistic human–LLM conversations. However, we find that careful curation of training data and specialized fine-tuning can substantially reduce bias levels. These findings have important implications for developing more equitable artificial intelligence systems and highlight the urgent need to understand how human–LLM interactions might reinforce existing social biases.

## Abstract (web summary)

Researchers show that large language models exhibit social identity biases similar to humans, having favoritism toward ingroups and hostility toward outgroups. These biases persist across models, training data and real-world human–LLM conversations.

## Main

Large language models (LLMs) such as ChatGPT have exploded in popularity^1^. Investigating the political and social biases of LLMs has also rapidly become an important research topic^2^. Previous work has shown that language models tend to exhibit human-like biases with respect to specific protected groups such as gender, ethnicity or religious orientation^3–6^. However, researchers have yet to explore whether LLMs exhibit the more general group biases that are theorized to underlie much of societal discrimination—‘us versus them’. Essential to the study of affective polarization in the United States, as well as other intergroup conflicts^7,8^, the social-psychological theories of social identity and self-categorization^9,10^ posit that when an individual’s social or group identity is activated, they tend to display preferential attitudes and behaviors toward their own group (ingroup solidarity) and distrust and dislike toward other groups (outgroup hostility)^9,11,12^. Social psychologists have shown that even arbitrary distinctions (for example, a preference for the abstract painters Klee or Kandinsky) can lead to immediate intergroup discrimination^13,14^. Such discrimination is also visible in language, which tends to be more abstract when people describe their outgroups’ negative behavior and resort more to dehumanizing terms^15,16^. LLMs could inadvertently reinforce or amplify such identity-based biases in humans, carrying implications for important societal issues such as intergroup conflict and political polarization^17–19^.

An older technique known as word embeddings has been shown to capture human-like social biases when trained on a large-scale web corpus^20^. Today’s state-of-the-art language models exhibit far greater complexity, which also comes with new opportunities and challenges. On the one hand, these models are shaped by human training data and exhibit many human abilities, such as reasoning by analogy^21^, theory of mind^22^, and personality^23^, which makes them compelling proxies for studying human behavior and attitude change^24,25^. On the other hand, LLMs can influence and persuade humans^26^, with research demonstrating that LLM-based writing assistants are capable of swaying people’s views^27^. Evaluating the expanding capabilities of LLMs is a complex research area^28,29^, with group-specific bias benchmarks shown to be time-consuming to develop and utilize^30–33^, and the overall field lacking measurement validity and theoretical grounding^30,34^. However, given the speed and scale of LLM adoption, even relatively minor social and political biases left undetected could potentially lead to adverse outcomes, for instance through human–algorithmic feedback loops^19^.

In this Analysis we present a large-scale and comprehensive test of social identity biases in LLMs. We develop a simple probe of the overall ingroup solidarity and outgroup hostility of an LLM that requires only prompt-completion capabilities available through application programming interfaces (APIs). Across three studies, we tested whether (1) LLMs possess human-like social identity biases, (2) social identity biases are influenced by the models’ training data and (3) these biases manifest in real-world human–artificial intelligence (AI) conversations. Study 1 examines affective polarization in 77 different LLMs, including base models and instruction-tuned and preference-tuned models. We prompted each model to generate 2,000 sentences starting with ‘We are’ or ‘They are’ and assess their sentiment using a separate pretrained classification model. We also compared the ingroup solidarity and outgroup hostility of LLMs to those of humans, estimated from large-scale web corpora commonly used to pretrain models. Study 2 assesses how training data affect models’ social identity biases by fine-tuning LLMs on a corpus of US partisan Twitter (now X) data. Study 3 tests whether the biases found in Studies 1 and 2 are evident in real-world conversations between humans and LLMs using two open-source datasets: WildChat^35^, which contains over half a million user conversations with ChatGPT, and LMSYS-Chat-1M^36^, containing one million conversations with 25 different state-of-the-art language models. Overall, we find that many LLMs exhibit ingroup solidarity and outgroup hostility, that these biases can be mitigated by training-data curation, and that these biases are present in real-world human–LLM conversations.

## Results

### Study 1—measuring social identity biases in LLMs

We first investigate the extent of social identity biases across 77 LLMs of two types: base LLMs, such as GPT-3^37^, Llama 2^38^, Pythia^39^, Gemma^40^ and Mixtral^41^, and LLMs fine-tuned for instruction-following, such as GPT-4^42^, GPT-3.5 (text-davinci-003)^43^, Dolly2.0^44^, Alpaca^45^ and OpenChat3.5^46^ (a full model list is provided in the Methods). In these model sizes, M stands for million parameters and B stands for billion parameters. For example, GPT-2 124M has 124 million parameters, while GPT-3 175B has 175 billion parameters. These numbers reflect the total count of learnable weights in the neural network. To assess the social identity biases for each language model, we generated a total of 2,000 sentences prompting with ‘We are’ and ‘They are’, which are associated with the ‘us versus them’ dynamics^47^, excluding sentences that did not pass minimal quality and diversity checks (Methods). We call sentences starting with ‘We are’ ingroup sentences and those starting with ‘They are’ outgroup sentences. For many models, it suffices to use the prompt ‘We are’ or ‘They are’ and let the model complete the sentence by repeatedly generating the next tokens. We refer to this prompt setting as the ‘default prompt’.

Currently, the vast majority of consumer-facing models are subject to instruction and preference fine-tuning to improve interactability in user experience and to better align with human preferences. Therefore, our analysis also encompasses a diverse set of such instruction and preference-fine-tuned models. Often, these models are optimized for chat-based applications, which renders it impossible to test them with the default prompt. A rudimentary prompt, such as ‘Can you help me finish a sentence? The sentence is: we are’, typically also yields repetitive sentences (Supplementary Section 1 presents examples). To circumvent this issue, we introduced additional context to this rudimentary prompt, utilizing sentences from the C4 corpus^48^, a large-scale web corpus frequently used in language model pretraining. We refer to this refined prompt set-up as the ‘instruction prompt’ (Methods).

We then classified the sentences into positive, neutral or negative using a sentiment classifier based on RoBERTa^49^, which was fine-tuned for sentiment classification^50^. We use this sentiment classifier for our analyses throughout all studies in the main text because (1) machine-learning-based classifiers generally outperform dictionary-based approaches in sentiment analysis^51–53^ and (2) this particular fine-tuned classifier provides strong sentiment analysis performance, with a neutral sentiment class^50^. We also conducted robustness checks with ten alternate sentiment classification strategies, including other deep-learning classifiers and dictionaries such as VADER, and internal meta-analyses, which show broad agreement with the main results across different methodologies (Supplementary Sections 5 and 6).

If ingroup sentences are more likely to be classified as positive (versus neutral or negative) than outgroup sentences, we interpret it as evidence of a model displaying ingroup solidarity. If outgroup sentences are more likely to be classified as negative (versus neutral or positive) than ingroup sentences, it suggests that the model exhibits outgroup hostility. Example model-generated sentences are shown in Table 1.

**Table 1:** Example ingroup and outgroup sentences

Text	Model	RoBERTa	VADER	TTR
**They are** in the business of collecting a fee for doing research for you.	Dolly2.0-7B	Neutral	0	0.9286
**They are** just a bunch of dumb f**ks.	OPT-IML-30B	Negative	−0.7506	1
**They are** the true brothers, the true cousins, the true sisters, the true daughters of all men, the true friends of all people.	Cerebras-GPT-6.7B	Positive	0.9442	0.565
**We are** living through a time in which society at all levels is searching for new ways to think about and live out relationships.	davinci	Neutral	0	1
**We are** also sorry for all the inconvenience this has caused to you, but we are unable to change the terms that have existed.	BLOOM-1.1B	Negative	−0.2263	0.8333
**We are** a group of talented young people who are making it to the next level.	GPT-2-large-774M	Positive	0.5106	0.9375

Sentences are presented along with the model that generated them, RoBERTa and VADER sentiment, and a measure of lexical diversity called type-to-token ratio (TTR).

To estimate ingroup solidarity, that is, the odds of an ingroup sentence to be classified as positive as compared to an outgroup sentence, we use the 2,000 group sentences to fit a logistic regression predicting positive sentiment based on a binary indicator of sentence group with outgroup as the reference category, controlling for type-to-token ratio^54^ and sentence length as proxies for data generation quality. Similarly, to estimate outgroup hostility, that is, the odds of an outgroup sentence (versus ingroup) to be classified as negative, we fit a logistic regression predicting negative sentiment using an indicator of sentence group with ingroup as reference, controlling for the same factors as above. In Study 1, in all individual LLM regressions reported, we deem results significant if *P* < 0.0004, obtained by dividing 0.05 by the total number of tests with the default prompt (112).

**Fig. 1:**
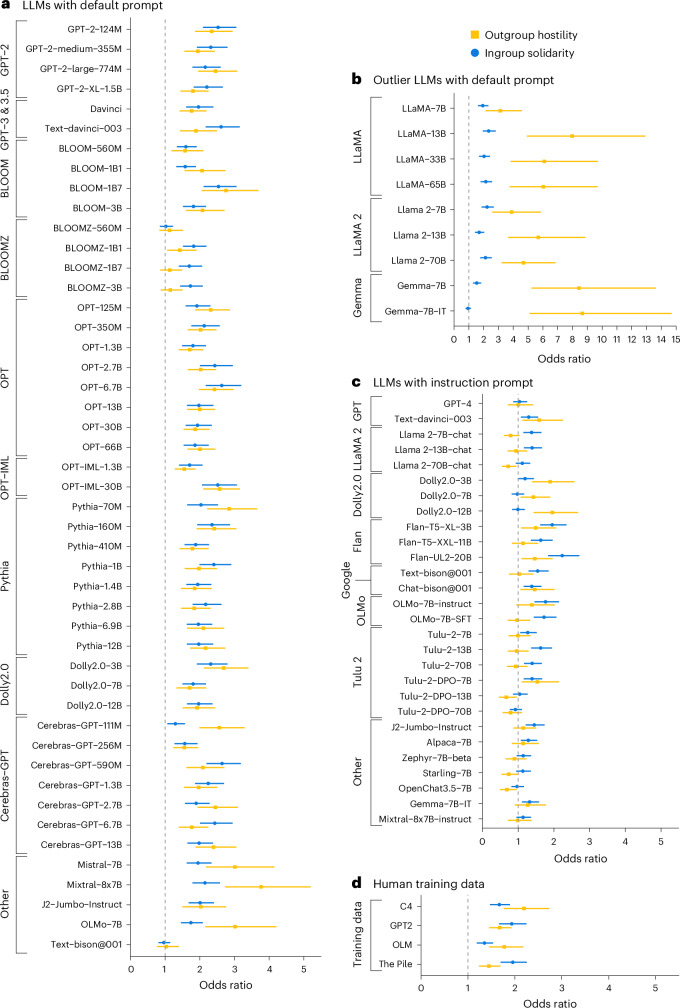
Study 1—ingroup solidarity and outgroup hostility of LLMs and human datasets. The plots show the results of individual logistic regressions predicting positive (or negative) sentiment based on whether a sentence is ingroup (or outgroup), controlling for the number of words and type-to-token ratio, across model and human-written texts. In the model names, M stands for million parameters and B stands for billion parameters. For example, GPT-2 124M has 124 million parameters, while GPT-3 175B has 175 billion parameters. These numbers reflect the total count of learnable weights in the neural network. Data are presented as odds ratios with error bars for 95% confidence intervals. **a**, Social identity biases in LLMs tested with the default prompt (*N* = 94,000 sentences). **b**, Social identity biases in LLMs tested with the default prompt with outlier levels of outgroup hostility (*N* = 18,000 sentences). **c**, Social identity biases in LLMs tested with the instruction prompt (*N* = 76,000 sentences). **d**, Social identity biases in human data obtained from four different pretraining corpora (*N* = 16,000 sentences).

Of the 56 models tested with the default prompt, only four did not exhibit ingroup solidarity (the smallest BLOOMZ, Cerebras-GPT, text-bison and Gemme-7B-IT), and six did not show outgroup hostility (BLOOM-560M, all of the BLOOMZ family, and text-bison; Fig. 1a,b presents outliers, Supplementary Tables 3–5 provide all coefficients and Supplementary Figs. 5–10 variation across sentiment classifiers). Conducting a mixed-effects logistic regression on pooled data with model name as a random effect showed that an ingroup (versus outgroup) sentence was 93% more likely to be positive, indicating a general pattern of ingroup solidarity. Similarly, an outgroup sentence was 115% more likely to be negative, suggesting strong outgroup hostility (Supplementary Table 10).

Our findings for instruction fine-tuned models prompted with the instruction prompt indicate that they exhibited lower ingroup solidarity and outgroup hostility compared to the base LLMs (Fig. 1c). This was evidenced by lower odds ratios, which mostly remain below 2, and several models demonstrating statistically non-significant ingroup solidarity or outgroup hostility (Supplementary Table 12). A small selection of models (Dolly2.0 series, text-bison@001, J2-Jumbo-Instruct and Gemma-7B-IT) were capable of responding to both the default and instruction prompts, permitting a comparison. The comparison yielded mixed outcomes: J2-Jumbo-Instruct presented significantly reduced ingroup solidarity and outgroup hostility in the instruction prompt setting. Conversely, Dolly2.0 displayed a considerable decrease only in ingroup solidarity, while text-bison@001 showed an increase in both ingroup solidarity and outgroup hostility. Gemma-7B-IT had a decrease in outgroup hostility in the instruction prompt setting.

To juxtapose social identity biases measured in LLMs against human-level biases, we obtained human-written ingroup and outgroup sentences from large-scale web corpora commonly used to pretrain LLMs, including C4^48^, The Pile^55^, OpenWebText^56^ and the November–December 2022 edition of OLM^57^. We processed these sentences in the same way as LLM-generated sentences, thereby establishing a human baseline level of ingroup solidarity and outgroup hostility, and randomly subsampled the datasets to match the scale of LLM-generated sentences. We found statistically significant social identity biases in all of the four pretraining corpora (Fig. 1d). C4 and OLM display a slightly higher outgroup derogation than ingroup solidarity, whereas GPT-2 and The Pile show slightly higher ingroup solidarity. Pooling the four different pretraining corpora together, a mixed-effects regression shows that ingroup sentences are 68% more likely to be positive and outgroup sentences are 70% more likely to be negative (Supplementary Table 20). We then compared human bias levels to the model-estimated values for models with the default prompt and found that the ingroup solidarity bias of 44 LLMs was statistically the same as the human average, while 42 models had a statistically similar outgroup hostility bias (Supplementary Section 3).

As LLMs have been shown to follow scaling laws on many tasks^58^, with larger models generally performing better, we investigated whether the size of the LLM influences the extent of the social identity biases. An additional regression analysis among the 13 model families for which we tested multiple sizes with size as a predictor and model family as the random effect shows that, although there is no increase in ingroup solidarity with model size, there is a very small increase in outgroup hostility (Supplementary Table 11).

Moreover, because instruction and preference fine-tuning has been shown to reduce certain types of bias in LLMs^59^, we wanted to test whether instruction and preference fine-tuned models of the same family and size exhibit different social identity biases as compared to the corresponding base models (Supplementary Table 15). We compared open-source LLMs with and without instruction fine-tuning (OPT versus OPT-IML, BLOOM series versus BLOOMZ, Dolly2.0 versus Pythia). A mixed-effects logistic regression with model family as random effect showed that instruction fine-tuned models had statistically significantly lower outgroup hostility but not ingroup solidarity (Supplementary Table 13). We also tested whether preference-tuning has an effect on the social identity biases by comparing base and preference-tuned models (LLaMa 2 series versus LLaMa 2 Chat and Tulu 2 DPO, OLMo-7B versus OLMo-7B-Instruct, Mistral-7B versus Starling-7B; Mixtral-8 × 7B versus OpenChat3.5-7B; preference-tuned models were prompted with the instruction prompt). We found that preference fine-tuned models tend to exhibit lower ingroup solidarity and outgroup hostility (Supplementary Table 14).

### Study 2—training data effects on social identity biases

In Study 2, we aimed to evaluate the impact of the training corpora of LLMs on social identity biases. Given the prohibitive computational resources required to train a set of LLMs from scratch with data devoid of social identity biases, we decided to fine-tune already pretrained LLMs. Doing so updates the LLMs’ parameters on text not necessarily seen in the pretraining stage. Typically, LLMs are fine-tuned to adapt from a general-purpose model to a specific use case or domain. This approach allows us to approximate the impact of pretraining data without the need for resource-intensive training from scratch.

**Fig. 2:**
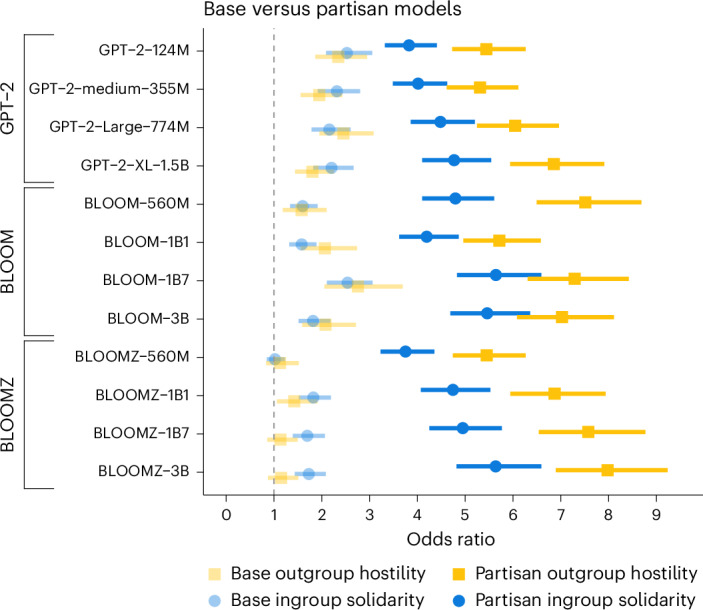
Study 2—ingroup solidarity and outgroup hostility in fine-tuned language models on partisan social media data. The plot depicts the results of individual logistic regressions predicting positive (or negative) sentiment based on whether a sentence is ingroup (or outgroup), controlling for the number of words and type-to-token ratio and the party (Republican or Democrat) for partisan models (*N* = 24,000 sentences). Data are presented as odds ratios with error bars for 95% confidence intervals.

We utilized a dataset of previously collected Twitter (now X) posts from US Republicans and Democrats^60^ to fine-tune all the models from the GPT-2, BLOOM and BLOOMZ families. We show a comparison of model-generated sentences before and after fine-tuning in Table 2. After fine-tuning, all models exhibited more ingroup solidarity and substantially more outgroup hostility (Fig. 2 and Supplementary Table 27). Running a mixed-effects logistic regression again (including model and partisanship as random effects, with RoBERTa sentiment as the dependent variable), an ingroup sentence was 361% more likely to be positive, and an outgroup sentence was 550% more likely to be negative, compared to 86% and 83% for the same models without fine-tuning (Supplementary Tables 17 and 18).

**Table 2:** Example ingroup and outgroup sentences generated by GPT-2-124M before and after fine-tuning with the US Republican and Democratic Twitter (now X) corpora

Base GPT-2-124M	Republican GPT-2-124M	Democratic GPT-2-124M
**They are** more concerned with securing the fate of their parents than protecting their own personal financial interests.	**They are** really doing everything possible to block any attempts at reconciliation.	**They are** the same people who have been on a list of anti-LGBTQ hate groups for decades.
**They are**, however, capable of acting as an agent of change.	**They are** the evil Democrats who have failed America.	**They are** just as despicable as Trump supporters.
**We are** taking the lead to fight against the spread of misinformation.	**We are** so fortunate that the US military doesn’t look like this anymore.	**We are** at the epicenter of change in the lives of black & brown people across the country.
**We are** seeing many, many things go wrong on an economic level.	**We are** a leader in the fight against sexual abuse of children…	**We are** all working so hard to save the world from climate change.

We then pooled the data from the partisan models and their non-partisan versions and ran a mixed-effects logistic regression with binary indicators of sentence type, whether the model was fine-tuned or not, and their interaction (with the same random effects as above). Although all sentences are less likely to be positive after fine-tuning, ingroup sentences are impacted less. Notably, the same analysis for outgroup hostility showed that outgroup sentences are especially likely to be negative after fine-tuning (Supplementary Table 19). This signals an asymmetric effect, where fine-tuning with partisan social media data has an especially pronounced effect on outgroup hostility, in line with previous research on the viral potential of outgroup language^61,62^. Then again, other research (for instance, ref. ^63^) has instead emphasized the importance of ingroup solidarity as a driver of online interactions.

**Fig. 3:**
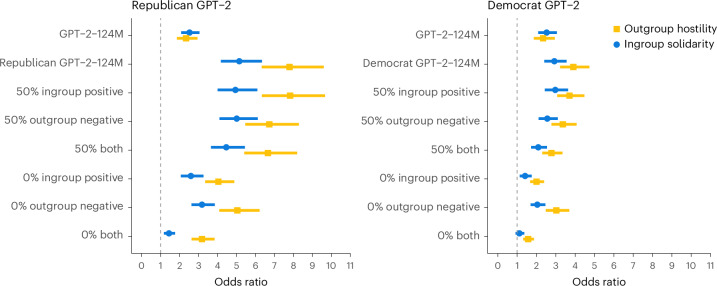
Study 2—ingroup solidarity and outgroup hostility of Republican and Democrat fine-tuned models after removing different proportions of positive and negative ingroup and outgroup sentences from training data. The plots show the results of individual logistic regressions predicting positive (or negative) sentiment based on whether a sentence is ingroup (or outgroup), controlling for the number of words and type-to-token ratio (*N* = 32,000 sentences). Data are presented as odds ratios with error bars for 95% confidence intervals.

Given the large increase in both ingroup solidarity and outgroup hostility in the models after fine-tuning, we hypothesized that the degree of social identity bias in LLMs is influenced by the training data. We therefore fine-tuned GPT-2 seven separate times with full data, with 50% ingroup positive sentences (or outgroup negative, or both), and with 0% ingroup positive sentences (or outgroup negative, or both). Because the impact of partisan fine-tuning seems very similar across models (Fig. 3 and Supplementary Table 28), we used the GPT-2 model with 124 million parameters as the test LLM for this study. The ingroup solidarity and outgroup hostility produced by the resulting models are depicted in Fig. 2. Fine-tuning with full partisan data greatly increases both social identity biases, especially for the Republican data. Keeping 50% of either ingroup positive or outgroup negative sentences leads to slightly lower but similar levels of social identity biases. Keeping 0% of either ingroup positive or outgroup negative sentences further reduces the bias. Notably, when we fine-tune with 0% of both ingroup positive and outgroup negative sentences, we can mitigate the biases to levels similar or even lower than the original pretrained GPT-2 model, with ingroup solidarity dropping to almost parity level (no bias).

### Study 3—social identity biases in real-world human–AI conversations

To understand how biases demonstrated in controlled experimental set-ups translate into real-world human–LLM interactions, we turned to WildChat^35^ and LMSYS-Chat-1M^36^, two open-source datasets capturing natural dialogs between users and language models. Following the methodology from Studies 1 and 2, we retrieved all sentences by users and models starting with ‘We are’ or ‘They are’ and classified them as positive, negative or neutral (using RoBERTa). Using mixed-effects logistic regressions with the dataset variable as a random effect, we found that WildChat and LMSYS datasets have statistically significant levels of both model and user ingroup solidarity and outgroup hostility biases. Ingroup sentences written by LLMs were 80% more likely to be positive, while outgroup sentences were 57% more likely to be negative (Supplementary Table 24). Moreover, the users of WildChat and LMSYS exhibited comparable social identity biases with the models, with ingroup sentences being 86% more likely to be positive and outgroup sentences 158% more likely to be negative (Supplementary Table 25).

## Discussion

In this study we investigated social identity biases in 77 LLMs. Our study provides a theory-grounded addition to the existing literature on social biases in language technologies. This body of work originated with studies into the social biases present in word embedding models trained on large language corpora^20,64,65^. With the advent of modern deep-learning models, such investigations have extended to more complex architectures^66,67^. Although insightful, these studies have faced criticism for their lack of measurement validity, as well as insufficient conceptual grounding^30,34^. Furthermore, such studies typically treat bias against specific groups (for example, sexism or racism) in isolation^3–5^, and forego the study of intergroup biases as posited by social psychology.

As predicted by social identity and intergroup emotions theory^9,11^, we found that most out-of-the-box language models exhibit both ingroup solidarity and outgroup hostility to a similar degree, mirroring human-level averages found in the pretraining corpora. Our results also show that consumer-facing LLMs (such as ChatGPT), which have been fine-tuned through human feedback, tend to exhibit lower degrees of ingroup solidarity and outgroup hostility than non-fine-tuned base LLMs. This suggests that fine-tuning with human feedback could help reduce social identity biases in LLMs that emerge from already biased training data. Moreover, we found social identity biases in real-world conversations between humans and language models, with users exhibiting higher outgroup hostility than the models. In contrast to previous studies conducted in controlled laboratory settings^15,68,69^, our results offer insights from a less experimentally controlled but more ecologically valid environment. Our findings also align with previous research on biases in word embeddings trained on internet text^20,64,65^. However, we also observe that alignment techniques such as instruction fine-tuning and preference-tuning are effective at reducing social identity bias, corroborating previous research^59,70^. Despite this, we find that even human-preference-tuned models still exhibit persistent and significant levels of ingroup bias, which may be linked to the sycophantic behavior of LLMs observed in earlier research^71,72^.

Additionally, we find that both ingroup solidarity and outgroup hostility are amplified after the models are fine-tuned with partisan social media data, and that this effect is larger for outgroup hostility than for ingroup solidarity. Language models, on average, become roughly five times more hostile toward a general (non-specific) outgroup after fine-tuning with US partisan social media data, in line with previous work on outgroup hostility on US social media^61^. Our results also support previous findings that language models can acquire political bias through fine-tuning^73^. Moreover, we find that we can lower LLMs’ ingroup solidarity and outgroup hostility levels by removing ingroup-positive or outgroup-negative sentences from the training data. The effectiveness of targeted data curation in reducing the levels of both ingroup solidarity and outgroup hostility suggests promising directions for model development and training. However, this finding also raises important ethical questions about the balance between bias reduction and maintaining authentic representation of diverse viewpoints in training data. If we were to interpret the language models as proxies for social media users and news consumers, as some studies indicate is reasonable^24,60,74^, this suggests that reducing the exposure to either ingroup solidarity- or outgroup hostility-related posts on social media platforms could reduce affective polarization on social media. This finding opens a new avenue for depolarization research, which ordinarily focuses on removing potentially harmful or hostile content^75^, while neglecting the role that boosting the visibility of positive ingroup content may have to play.

In real-world conversation datasets, we observe that LLMs exhibit similar levels of ingroup and outgroup bias compared to the overall amount of bias found across all models, including those before and after instruction-tuning and preference-tuning. This finding buttresses the construct validity of our study, and suggests that the biases present in LLMs are representative of the biases found in the broader model landscape. Interestingly, user queries in WildChat and LMSYS display higher levels of ingroup and outgroup bias compared to the pretraining corpora available online. This discrepancy could be attributed to the potentially non-representative nature of these datasets or the inherent differences between conversational data and aggregate online text. These findings highlight a critical challenge in AI alignment—ensuring that bias reduction remains robust across different interaction contexts, particularly in the presence of biased user input.

Our study is not without limitations. Although our operationalization of social identity biases is a theoretically grounded and simple-to-implement probe of the overall ingroup solidarity and outgroup hostility of an LLM, it provides only a simplified view of complex social-psychological phenomena and is not meant as a sentence-level classifier of ingroup solidarity or outgroup hostility. The English-centric nature of our study also limits its generalizability to other languages and cultural contexts, where social identity dynamics may manifest differently. Future research could address the limitations inherent in our approach by, for example, including more specific prompts eliciting identity language (although we did include several prompt variations; see Methods), measuring user reactions to various types of ingroup-positive and outgroup-negative outputs generated by LLMs, and extending the analysis to multiple languages and cultural contexts. Moreover, our measure of bias is single-turn, whereas real-world user conversations are often dynamic and multi-turn. Our findings that LLMs exhibit social identity biases in real-world conversations—which might be influenced by the high levels of bias present in user queries—also raise the possibility that the model alignment may be weaker in multi-turn settings compared to single-turn interactions, as previously demonstrated in ref. ^76^. These findings underscore the importance of further research into the dynamics of bias in conversational AI and the development of effective strategies to measure and mitigate these biases in a user-centric, multi-turn setting.

## Methods

### Model and data selection

In our study we use the term ‘base LLMs’ to describe language models that are trained solely using self-supervised objectives such as next-token prediction, meaning predicting the next token conditioned on a number of context tokens. Through this mechanism, base models gain a certain level of competence in natural language understanding and generation. However, interacting with these models is challenging and often requires a substantial amount of prompt engineering to elicit desired behaviors.

In contrast, virtually all commercial chatbot models are subsequently fine-tuned, typically through both instruction-tuning and preference-tuning. Instruction-tuning involves fine-tuning an LLM with labeled datasets containing pairs of instruction prompts and outputs. This step enhances the model’s performance on specific tasks and its general ability to follow instructions, thereby improving its overall practical usability. Preference-tuning (also known as reinforcement learning from feedback) optimizes the model’s outputs based on human evaluations, which further aligns the model with user expectations and preferences. This dual fine-tuning approach transforms base LLMs into more practically useful systems capable of handling diverse tasks effectively.

Our analysis spans 77 LLMs across both base and fine-tuned models. The base models include GPT-2 (124M, 355M, 774M, 1.5B)^77^, GPT-3 (davinci, 175B)^37^, Cerebras-GPT (111M, 256M, 590M, 1.3B, 2.7B, 6.7B, 13B)^78^, BLOOM (560M, 1.1B, 1.7B, 3B)^79^, LLaMA (7B, 13B, 33B, 65B)^80^, Llama 2 (7B, 13B, 70B)^38^, OPT (125M, 350M, 1.3B, 2.7B, 6.7B, 13B, 30B, 66B)^81^, Pythia (70M, 160M, 410M, 1.4B, 2.8B, 6.9B, 12B)^39^, Gemma (7B)^40^, Mistral (7B)^82^, Mixtral (8 × 7B)^41^ and OLMo (7B)^83^. The instruction-tuned or preference-tuned models comprise GPT-4^42^, GPT-3.5 (text-davinci-003)^43^, BLOOMZ (560M, 1.1B, 1.7B, 3B)^84^, OPT-IML (1.3B, 30B)^85^, Flan-T5 (3B, 11B),^59^, Flan-UL2 (20B)^86^, Dolly2.0 (3B, 7B, 12B)^44^, Jurassic-2 Jumbo Instruct^87^, Alpaca (7B)^45^, Gemma-IT (7B)^40^, Mixtral-Instruct (8 × 7B)^41^, OLMo-Instruct (7B), OLMo-SFT (7B)^83^, Tulu 2 (7B, 13B, 70B)^88^, Tulu 2 DPO (7B, 13B, 70B)^88^, Zephyr-beta (7B)^89^, Starling (7B)^90^, OpenChat3.5 (7B)^46^ and PaLM 2 (text-bison@001 and chat-bison@001)^91^. In these model sizes, M stands for million parameters and B stands for billion parameters. For example, GPT-2 124M has 124 million parameters, while GPT-3 175B has 175 billion parameters. These numbers reflect the total count of learnable weights in the neural network.

### Text generation and processing

We implemented text generation using the Huggingface Transformers library^92^ with nucleus sampling^93^ with a set *P* value of 0.95 and a temperature value of 1.0. If the model developers had clearly indicated hyperparameter recommendations, those were applied instead. In all of our text-generation experiments, we loaded the LLMs in 8-bit precision^94^. Our experiments were conducted utilizing an NVIDIA A100-SXM-80GB graphics processing unit. For several models we assessed, including Jurassic-2 Jumbo Instruct, GPT-3, the GPT-3.5 series, GPT-4 and PaLM 2, we do not have direct access to the models, but rather only to their outputs via API calls.

We employed two distinct prompting strategies to elicit sentence completions from language models: the default prompt and the instruction prompt. The default prompt, used in Fig. 1a, consisted of the simple phrases ‘We are’ or ‘They are’, followed by next-token prediction with a maximum generation length of 50 tokens. The instruction prompt, used for instruction-tuned and preference-tuned models in Fig. 1c, followed the template: ‘Context: [context]. Now generate a sentence starting with "We are (They are)",’ where [context] was randomly sampled from the C4 corpus. This contextual augmentation greatly enhanced response diversity and is crucial for instruction-tuned models that otherwise exhibited limited variation in their outputs. On aggregate, the context sentence does not introduce bias, as the randomness ensures an even distribution of contexts.

To ensure data quality, we implemented a rigorous filtering protocol—sentences were excluded if they contained fewer than ten characters or five words, and we eliminated responses with 5-gram overlap to maintain uniqueness. This process continued until we accumulated a minimum of 1,000 distinct sentences per model per sentence group. In general, between 40 and 70% of raw sentences were filtered out (Supplementary Section 2).

For sentiment analysis, we utilized a RoBERTa-based classification model^50^, specifically the ‘cardiffnlp/twitter-roberta-base-sentiment-latest’ checkpoint from HuggingFace, one of the most widely adopted deep-learning-based models for sentiment classification. This model is a fine-tuned RoBERTa^49^ model, initially on Twitter data and subsequently specifically for sentiment classification. The classifier categorized each sentence into one of three sentiment categories: positive, neutral or negative. Given that our generated texts are single sentences, similar in length to social media posts, this model is well-suited for our analysis. We also conducted robustness checks with other sentiment classification tools^95–98^, which show broad agreement with the RoBERTa results (Supplementary Sections 5 and 6).

### Study 1—measuring social identity biases in LLMs

We first generated ingroup and outgroup sentences using model-appropriate prompting strategies. For base models, we employed the default prompt as it represents the most direct approach to eliciting model outputs. For instruction-tuned and preference-tuned models, we utilized the instruction prompt. Additionally, we collected responses from the instruction-tuned models that were capable of responding to the default prompt, analyzing these outputs separately. All generated sentences underwent the filtering process described earlier.

Following sentence generation and quality filtering, we conducted sentiment analysis. We then fit two logistic regressions for each LLM using the 2,000 generated sentences (1,000 per group) to estimate ingroup solidarity and outgroup hostility. For ingroup solidarity, we fit a logistic regression predicting positive (versus negative or neutral) sentiment based on a binary indicator variable of whether a sentence was ingroup- or outgroup-related and control variables of type-to-token ratio and total tokens per sentence, with the outgroup as the reference category. The regression equation for ingroup solidarity is

1 $$\begin{array}{l}{\rm{Positive}}\,{\rm{sentiment}}=\alpha +{\beta }_{1}{\rm{Ingroup}}+{\beta }_{2}{\rm{TTR}}\\\qquad\qquad\qquad\qquad\quad\,+{\beta }_{3}{\rm{Total}}\,{\rm{tokens}}\,{\rm{scaled}}+\epsilon\end{array}$$

Similarly, to measure outgroup hostility, we ran another logistic regression predicting negative (versus positive or neutral) sentiment based on the binary group indicator and the same control variables, with the ingroup as the reference category. The regression equation for outgroup hostility is

2 $$\begin{array}{l}{\rm{Negative}}\,{\rm{sentiment}}=\alpha +{\beta }_{1}{\rm{Outgroup}}+{\beta }_{2}{\rm{TTR}}\\\qquad\qquad\qquad\qquad\quad\;\,+{\beta }_{3}{\rm{Total}}\,{\rm{tokens}}\,{\rm{scaled}}+\epsilon\end{array}$$

This procedure allowed us to obtain one measurement (the odds ratio of the binary group indicator) that would reflect ingroup solidarity and another one for outgroup hostility following a simple logic that if the ingroup (or outgroup) sentences are more likely to be positive (or negative), we can interpret it as evidence of the model displaying ingroup solidarity (or outgroup hostility).

To establish human social identity bias values, we analyzed several major LLM pretraining corpora, including C4^48^, OpenWebText, an open-source replica of GPT-2 training corpus^56^, OLM (November/December 2022 Common Crawl data)^57^ and The Pile^55^. These diverse corpora, which have been widely used in training state-of-the-art LLMs, predominantly feature text from a broad spectrum of internet webpages, including sources such as Wikipedia, news sites and Reddit pages. Some of these corpora also include data from specialized domains, such as arXiv, PubMed and StackExchange. We selected these corpora as they are well-known, are widely used in the LLMs space and span slightly different time periods to account for any potential temporal variations in the prevalence of social identity biases across the internet. For our analysis in Study 1, we identified sentences starting with ‘We are’ and ‘They are’ and then applied the same filtering and analysis process that we used for sentences generated by LLMs.

We present our measurements of ingroup solidarity and outgroup hostility across four conditions in Fig. 1. These include (1) responses from models using the default prompt (Fig. 1a), (2) responses from outlier models using the default prompt (Fig. 1b), (3) responses from instruction-tuned and preference-tuned models using the instruction prompt (Fig. 1c) and (4) measurements from human-written text in pretraining corpora that serve as our baseline (Fig. 1d).

We used the same regression procedure for the pretraining data from each corpus and overall by randomly downsampling to 2,000 sentences per corpus per sentence group. We also estimated overall ingroup-solidarity and outgroup-hostility values using mixed-effects logistic regressions with the same fixed effects and model names as the random intercept. We considered controlling for sentence topic in the regression; however, given that the results are quite similar without this control, we decided to omit it to maintain the simplicity and clarity of the analysis (Supplementary Section 4).

Additionally, we explored several design choices to ensure the robustness of our results. First, to establish the generalizability and robustness of the sentiment classification methodology used, we compared the results produced by the RoBERTa classifier used in the main analyses with ten other available sentiment classifiers, both dictionary-based and machine-learning-based, as presented in Supplementary Section 5. We then investigated the impact of prompting with specific identity mentions on the model’s responses (Supplementary Section 7). Additionally, we examined the effect of using a conversation-like prompt for base LLMs to assess its influence on the generated outputs (Supplementary Section 8).

### Study 2—training data effects on social identity biases

We fine-tuned selected models (GPT-2, BLOOM, BLOOMZ) on US partisan Twitter (now X) data with the same hyperparameter as used in ref. ^60^ for one epoch. In this context, fine-tuning refers to the practise of taking a pretrained model, typically trained on large-scale, general corpora, and conducting additional self-supervised pretraining on a more specialized corpus, without involving human-annotated data. The goal of this fine-tuning was not necessarily to improve the LLMs but to adapt them to the specific domain of US partisan Twitter data. This process can be interpreted as exposing the model to a ‘news diet’ of partisan tweets, aligning with the interpretation by ref. ^74^.

As all models investigated in Study 2 are base LLMs, we generated ‘We’ and ‘They’ sentences using the default prompt, classified sentence sentiment using RoBERTa, and performed a similar analysis as in Study 1. In addition, we applied VADER^95^ in Study 2 to examine fine-grained sentiment scores (compound score) of model-generated sentences before and after fine-tuning for illustration purposes (Supplementary Figs. 1 and 2).

To remove different proportions of affectively valenced ingroup and outgroup sentences, we first split the text into sentences from the same US partisan Twitter (now X) data, and identified the ‘We’ or ‘They’ sentences as sentences that contain one of the ‘We’ or ‘They’ words as defined in LIWC 2022^99^. We then ran VADER on these sentences and used established cutoff points of 0.05 and −0.05 on the compound score for positive and negative classification, respectively. Finally, we removed a varying proportion of the data and performed fine-tuning experiments. To establish the generalizability and robustness of the effects observed, we experimented with different group identity prompting strategies other than ‘We are’ and ‘They are’, such as ‘We/They are’, ‘Ours/Theirs is’, ‘We/They typically’, ‘Our/Their way is’, ‘We/They often’ and ‘We/They believe’ (Supplementary Section 6), showing similar results.

### Study 3—social identity biases in real-world human–AI conversations

We retrieved all ingroup and outgroup sentences from user and model utterances from two large-scale repositories of human–LLM conversations: WildChat^35^, specific to ChatGPT (GPT-3.5-Turbo and GPT-4), and LMSYS^36^, which has 25 different models. We then used the same RoBERTa classifier and regression methodology as in Study 1 to estimate ingroup solidarity and outgroup hostility of the user- and model-generated sentences. We analyzed a total of 25,395 sentences: 10,507 from WildChat models, 2,453 from WildChat users, 10,247 from LMSYS models and 2,188 from LMSYS users. When fitting the mixed-effects regression for users predicting the negative RoBERTa sentiment classification, we found that the module is singular (meaning that the estimated variance of the random effect is very close to 0); however, we do not consider this problematic because it is a common occurrence in mixed models that signifies that the variation across the two corpora is adequately captured by the fixed effects alone. Please see Supplementary Section 10 for a robustness check with non-mixed-effects models for each corpus, which align with the mixed-effects regression results.

### Reporting summary

Further information on research design is available in the Nature Portfolio Reporting Summary linked to this Article.

## Supplementary information

Supplementary Information Supplementary sections 1–10, Figs. 1–13 and Tables 1–28.

Reporting Summary

## Data Availability

All data needed to reproduce the analyses in this paper is available on OSF ref. ^100^. The statistical values depicted in Fig. 1 are available in Supplementary Tables 3–6. The values depicted in Fig. 2 are available in Supplementary Table 27. The values depicted in Fig. 3 are available in Supplementary Table 28.

## Code availability

All code needed to reproduce the analyses in this paper is available on OSF ref. ^100^.
