# Supplementary material for: Generative language models exhibit social identity biases
Source: Nat Comput Sci. 2024 Dec 12;5(1):65–75. doi: 10.1038/s43588-024-00741-1 (PMC11774750; doi:10.1038/s43588-024-00741-1)
Supplement: Supplementary file 1 — Supplementary sections 1–10, Figs. 1–13 and Tables 1–28. [file 43588_2024_741_MOESM1_ESM.pdf]

nature computational science

Supplementary information https://doi.org/10.1038/s43588-024-00741-1

Generative language models exhibit social identity biases

In the format provided by the authors and unedited

Table of Contents

1. Supplementary Text *4*

1.1. Study 1: Repetitive sentence output using a rudimentary prompt on instruction-tuned models 4

1.2. Study 1: Effect of sentence filtering 4

1.3. Study 1: Difference between model and human data 4

1.4. Study 1: Controlling for sentence topic with a Structural Topic Model 5

1.5. Study 1, 2, 3: Sentiment classifiers 5

1.6. Study 1, 2: Ingroup and outgroup prompting formulations 6

1.7. Study 1: Exploring specific identities 6

1.8. Study 1: Conversational prompt for base models 6

1.9. Study 2: Partisan finetuning with different proportions of ingroup positive and outgroup negative sentences 7

1.10. Study 3: Logistic regressions 7

2. Supplementary Figures *8*

2.1. Supplementary Figure 1: Study 2: VADER sentiment scores for ingroup and outgroup sentences generated by BLOOM 1.1B, before and after fine-tuning on Republican or Democrat Twitter (now X) data. 8

2.2. Supplementary Figure 2: Study 2: VADER sentiment score of model-generated sentences across fine-tuning settings. 9

2.3. Supplementary Figure 3: Study 1: Diagnostic metrics for structural topic models across varying numbers of topics. 10

2.4. Supplementary Figure 4: Study 1: Structural topic modeling analysis of model-generated sentences. 11

2.5. Supplementary Figure 5: Study 1: Logistic regression analysis across language models tested with the default prompt with different sentiment classifiers. 12

2.6. Supplementary Figure 6: Study 1: Logistic regression analysis across language models tested with the instruction prompt with different sentiment classifiers. 13

2.7. Supplementary Figure 7: Study 1: Logistic regression analysis across pertaining corpora with different sentiment classifiers. 14

2.8. Supplementary Figure 8: Study 2: Logistic regression analysis for BLOOM-560M and BLOOMZ-560M with no or partisan fine-tuning and different prompting formulations and sentiment classifiers. 15

2.9. Supplementary Figure 9: Study 2: Logistic regression analysis for GPT2-124M with none, partisan, or curated (ingroup and outgroup sentences removed) fine-tuning and with different prompting formulations and sentiment classifiers. 16

2.10. Supplementary Figure 10: Study 3: Logistic regression analysis of human-LLM conversations with different sentiment classifiers. 17

2.11. Supplementary Figure 11: Study 1: Logistic regression analysis of GPT-2-124M when mentioning specific group identities. 18

2.12. Supplementary Figure 12: Study 1: Logistic regression analysis of language models tested with an alternative conversation-like prompt. 19

2.13. Supplementary Figure 13: Study 2: Logistic regression analysis of the effects of different training data compositions on finetuning outcomes. 20

3. Supplementary Tables 21

3.1. Supplementary Table 1: Study 1: Ratio of sentences kept post filtering (default prompt). 21

3.2. Supplementary Table 2: Study 1: Ratio of sentences kept after filtering (instruction prompt).. 22

3.3. Supplementary Table 3: Study 1: Ingroup solidarity and outgroup hostility of LLMs tested with the default prompt (Part 1).. 23

3.4. Supplementary Table 4: Study 1: Ingroup solidarity and outgroup hostility of LLMs tested with the default prompt (Part 2).. 24

3.5. Supplementary Table 5: Study 1: Ingroup solidarity and outgroup hostility of outlier LLMs tested with the default prompt. 25

3.6. Supplementary Table 6: Study 1: Ingroup solidarity and outgroup hostility of pre-training datasets using a Random Subsample.. 26

3.7. Supplementary Table 7: Study 1: Ingroup Solidarity and Outgroup Hostility of Pre-training Datasets using the Full Datasets.. 27

3.8. Supplementary Table 8: Study 1: Ingroup solidarity and outgroup hostility of a subset base LLMs controlling for sentence topic (Part 1). 28

3.9. Supplementary Table 9: Study 1: Ingroup solidarity and outgroup hostility of a subset base LLMs controlling for sentence topic (Part 2). 29

3.10. Supplementary Table 10: Study 1: Mixed effects logistic regression results showing overall social identity biases for models tested with the default prompt.. 30

3.11. Supplementary Table 11: Study 1: Mixed effects logistic regression results showing the effect of model size on social identity biases. 31

3.12. Supplementary Table 12: Study 1: Ingroup solidarity and outgroup hostility of LLMs tested with the instruction prompt. 32

3.13. Supplementary Table 13: Study 1: Mixed effects logistic regression results showing the effects of instructions fine-tuning on social identity biases tested with the default prompt. 33

3.14. Supplementary Table 14: Study 1: Mixed effects logistic regression results showing the effects of preference fine-tuning on social identity biases tested with the instruction prompt. 34

3.15. Supplementary Table 15: Correspondence between base models, instruction-tuned models, and preference-tuned models. 35

3.16. Supplementary Table 16: Sentiment analysis results across three classifiers with and without swapping “We” for “They” and vice versa. 36

3.17. Supplementary Table 17: Study 2: Mixed effects logistic regression results showing overall social identity biases for the partisan fine-tuned models. 37

3.18. Supplementary Table 18: Study 2: Mixed effects logistic regression results showing overall social identity biases for base models before partisan fine-tuning. 38

3.19. Supplementary Table 19: Study 2: Mixed effects logistic regression results showing the effect of partisan fine-tuning. 39

3.20. Supplementary Table 20: Study 1: Mixed effects logistic regression results showing the overall ingroup solidarity and outgroup hostility biases of sentences from pre-training corpora. 40

3.21. Supplementary Table 21: Study 1: Logistic regression results showing the comparison of ingroup and outgroup bias of sentences from pre-training corpora and sentences from LLMs combined. 41

3.22. Supplementary Table 22: Study 1: Raw counts of positive and negative valanced ingroup and outgroup sentences for models tested with the default prompt. 42

3.23. Supplementary Table 23: Study 1: Descriptive statistics of various metrics for sentences generated with the default prompt. 43

3.24. Supplementary Table 24: Study 3: Mixed effects logistic regressions results showing social identity biases of LLM-generated sentences in real-world human-LLM conversations. 44

3.25. Supplementary Table 25: Study 3: Mixed effects logistic regressions results showing social identity biases of user-written sentences in real-world human-LLM conversations. 45

3.26. Supplementary Table 26: Study 3: Ingroup solidarity and outgroup hostility of both user-written and LLM-generated sentences in real-world human-LLM conversations. 46

3.27. Supplementary Table 27: Study 2: Ingroup solidarity and outgroup hostility of LLMs fine-tuned with partisan Twitter data. 47

3.28. Supplementary Table 28: Study 2: Ingroup solidarity and outgroup hostility of LLMs fine-tuned with curated partisan Twitter data. 48

1. Supplementary Text

1.1. Study 1: Repetitive sentence output using a rudimentary prompt on instruction-tuned models

As mentioned in Study 1, for many instruction-tuned models, especially the ones additionally preference-tuned to be chat bots, we cannot use the default prompt by simply supplying the model with “We(They) are” and expect the model to finish the sentence. A typical response from GPT-4 is “Sorry, it seems like your message got cut off. Can you please provide more context or finish your sentence so I can assist you?” A rudimentary attempt to rewrite this prompt into an instruction format “Can you help me finish a sentence? The sentence is: we are” typically also yields very repetitive sentences. We list 10 random sentences following this prompt below:

1. We are a team committed to achieving our goals and making a positive impact.
2. We are ready to tackle any challenge that comes our way.
3. We are ready to take on any challenge that comes our way.
4. We are always ready to assist you.
5. We are here to assist you with any questions or tasks you may have.
6. We are ready to face any challenge that comes our way. 7. We are fortunate to have such a supportive community.
8. We are ready to take on this challenge and overcome any obstacles in our way.
9. We are ready to tackle any challenge that comes our way.
10. We are here to assist you with any questions or concerns you may have.

This issue is not resolved by increasing the temperature. Therefore, we have to resort to the instruction prompt “Context: context. Now generate a sentence starting with ‘We are (They are)”’ where context was a random sentence from the C4 corpus.

1.2. Study 1: Effect of sentence filtering

In the preliminary step after sentence generation from the LLMs, we implement sentence filtering. This involves eliminating sentences with fewer than 10 characters or 5 words, and filtering out sentences with 5-gram overlap. Supplementary Tables 1 and 2 present the proportion of sentences retained post-filtration for each of the 51 models tested with the default prompt and the 15 models tested with the instruction prompt for which data was collected before September 2023. The proportion of retained sentences can be considered as a measure of the diversity in the sentences generated by each model. For the Default Prompt, the majority of models display a sentence survival rate of 30-40% for “we” sentences and 40-50% for “they” sentences. However, it is important to note that these rates vary considerably across different models. For the instruction prompt, the success rate is generally higher, with sentence survival rates reaching up to 70% for “we” sentences and 80% for “they” sentences. We attribute this elevated success rate for the instruction prompt to two primary factors: the models capable of accommodating the instruction prompt are typically larger and more advanced; secondly, providing a random context sentence encourages the model to generate more diverse outputs.

1.3. Study 1: Difference between model and human data

For a given model, we determined whether there was a significant difference ($p >= .0004$) in ingroup solidarity or outgroup hostility from human values by a logistic regression focusing on the interaction term of sentence group (ingroup or outgroup) and sentence origin (human or model). To make the scale of human data match the scale of model data we randomly downsampled to 4,000 sentences per corpus. For instance, to determine the difference between human and model ingroup solidarity, we used the p-value associated with β_3 in the equation below.

$$PositiveSentiment = \alpha + \beta_1 Ingroup + \beta_2 Human + \beta_3 Ingroup * Human$$
$$+ \beta_4 TTR + \beta_5 TotalTokensScaled + \epsilon$$

Ingroup Solidarity.

No significant difference: GPT-2-Medium-355M, GPT-2-Large-774M, GPT-2-XL1.5B, davinci, BLOOM-560M, BLOOM-1B1, BLOOM-3B, BLOOMZ-1B1, BLOOMZ1B7, BLOOMZ-3B, LLaMA-7B, LLaMA-30B, LLaMA-65B, Llama 2-7B, Llama 2-13B, Llama 2-70B, OPT-125M, OPT-350M, OPT-1.3B, OPT-13B, OPT-30B, OPT66B, OPT-IML-1.3B, Pythia-70M, Pythia-160M, Pythia-410M,

Pythia-1B, Pythia1.4B, Pythia-2.8B, Pythia-6.9B, Pythia-12B, Dolly2.0-3B, Dolly2.0-7B, Dolly2.012B, Cerebras-GPT-111M, Cerebras-GPT-256M, Cerebras-GPT-1.3B, CerebrasGPT-2.7B, Cerebras-GPT-13B, Gemma-7B, Mistral-7B, Mixtral-8x7B, J2-JumboInstruct, OLMo-7B.

Significant difference: GPT-2-124M, text-davinci-003, BLOOM-1B7, BLOOMZ560M, LLaMA-13B, OPT-2.7B, OPT-6.7B, OPT-IML-30B, Cerebras-GPT-590M, Cerebras-GPT-6.7B, Gemma-7B-IT, text-bison@001.

Outgroup Hostility.

No significant difference: GPT-2-124M, GPT-2-Medium-355M, GPT-2-Large774M, GPT-2-XL-1.5B, davinci, text-davinci-003, BLOOM-560M, BLOOM-1B1, BLOOM-3B, BLOOMZ-560M, BLOOMZ-1B1, BLOOMZ-1B7, BLOOMZ-3B, LLaMA-7B, OPT-125M, OPT-350M, OPT-1.3B, OPT-2.7B, OPT-6.7B, OPT13B, OPT-30B, OPT-66B, OPT-IML-1.3B, Pythia-160M, Pythia-410M, Pythia-1B, Pythia-1.4B, Pythia-2.8B, Pythia-6.9B, Pythia-12B, Dolly2.0-7B, Dolly2.0-12B, Cerebras-GPT-111M, Cerebras-GPT-256M, Cerebras-GPT-590M, Cerebras-GPT1.3B, Cerebras-GPT-2.7B, Cerebras-GPT-6.7B, Cerebras-GPT-13B, Mistral-7B, J2-Jumbo-Instruct, text-bison@001.

Significant difference: BLOOM-1B7, LLaMA-13B, LLaMA-30B, LLaMA-65B, Llama 2-7B, Llama 2-13B, Llama 2-70B, OPT-IML-30B, Pythia-70M, Dolly2.0-3B, Gemma-7B, Gemma-7B-IT, Mixtral-8x7B, OLMo-7B.

1.4. Study 1: Controlling for sentence topic with a Structural Topic Model

We fit a structural topic model as implemented in the R package 'stm' on all the sentences produced a subset of non-finetuned models, for which we collected the data before September 2023 (51 models). We also tried BERTopic for topic modeling, but it produced very poor results likely due to the short length of our texts. BERTopic also risked contaminating the results with the biases from the word embeddings. As we had to define the stm parameter K, which is the number of topics, we first fit four different stm models with values of K=20, 40, 60, and 80. Judging by the held-out likelihood (see Supplementary Figure 3), 60 was the best number of topics for this corpus. Therefore, we fit a new stm with K=60 and conduct further analyses with the resultant topics (see Supplementary Figure 4a). We found that there were significant differences between the topics of the ingroup and the outgroup sentences (see Supplementary Figure 4b). As a robustness check, we included the topic classification of a given sentence into the regression models that produce the ingroup solidarity and outgroup hostility coefficients as a control variable. For instance, for ingroup solidarity, the regression formula would be:

$$PositiveSentiment = \alpha + \beta_1 Ingroup + \beta_2 Topic + \beta_3 TTR + \beta_4 TotalTokensScaled + \epsilon$$

We found that the effects remain largely the same when controlling for the topic (see Supplementary Tables 8 and 9). Out of the 51 tested models, 45 exhibited ingroup solidarity with an average odds ratio of around 2.09, and 38 showed outgroup hostility with an average odds ratio of about 2.33.

1.5. Study 1, 2, 3: Sentiment classifiers

To establish the generalizability and robustness of the sentiment classification methodology used, we compared the results produced by the RoBERTa classifier used in the main analyses with other available sentiment classifiers. In addition to RoBERTa, we used DynaSent (v0) and GPT-4o-mini (the latter only for the default prompt in Study 1) and dictionary methods such as VADER [95], AFINN [98], TextBlob [99], and LIWC [96].

We also examined how swapping "We" with "They" and vice versa changes the sentiment of the deep learning methods (RoBERTa, DynaSent, and GPT-4o-mini) on a random sample of 10,000 "We" and 10,000 "They" sentences from Study 1. We found that the resulting proportions of positive, negative, and neutral sentences change depending on whether the sentence starts with "We" or "They" when the rest of the sentence remains unchanged, such that the models more often rate "We" sentences as positive (see Supplementary Table 16). However, the effect is small for GPT-4o-mini.

We additionally included the results of RoBERTa and DynaSent classifiers when evaluating sentiment with the "We" or "They" swapped (referred to as RoBERTa Swapped and DynaSent Swapped). This swapping approach artificially induces an anti-ingroup-outgroup bias, resulting in a smaller observed effect size than the true effect. We also included results when selecting the sentiment label with the highest probability from the non-swapped and swapped options (referred to as RoBERTa Pooled and DynaSent Pooled). For example, the sentence "We are ready to do this" gets a RoBERTa label of positive with a probability of .67, while the swapped sentence "They are ready to do this" receives a neutral label with a

probability of .8, resulting in both RoBERTa Swapped and RoBERTa Pooled labels for the original sentence being neutral. The pooled results could be seen as approximating the true effect size more closely, as the bias in the default model is counterbalanced by the anti-ingroupoutgroup bias of the swapped model.

Thus, in total, we used ten alternative sentiment classification strategies (excluding the RoBERTa model utilized throughout the main text). We used the same methodology as in the main analyses, namely logistic regressions shown in equations 1 and 2, to estimate the extent of ingroup solidarity and outgroup hostility, respectively, per LLM or corpus. We also include an estimated average odds ratio across the classifiers (excluding the Pooled classifiers as they are not independent from their non-Pooled versions) using random-effects meta-analysis models with restricted maximum likelihood estimator as implemented in the metafor R package, represented as "Meta Average (Excl. Pooled)" in the results plots. The results–which can be seen in the Supplementary Figures 5, 6, and 7 for Study 1 and Supplementary Figures 8 and 9 for Study 2 (the "We/They are" line), and Supplementary Figure 10 for Study 3–show broad agreement with the main conclusions of the paper across different methodologies.

1.6. Study 1, 2: Ingroup and outgroup prompting formulations

To establish the generalizability and the robustness of the effects observed in the studies, we experimented with different group identity prompting strategies other than "We are" and "They are" such as: "We/They are", "Ours/Theirs is", "We/They typically", "Our/Their way is", "We/They often", and "We/They believe".

We used the same methodology as in Study 1 (fitting separate logistic regressions for each LLM) and evaluated the sentiment using ten different classifiers (see A.5). The results broadly agree with the main conclusions of the paper (see Supplementary Figure 8 for BLOOM and BLOOMZ fine-tuning results and Supplementary Figure 9 for GPT2 fine-tuning and data curation results). Specifically, the measures of ingroup solidarity and outgroup hostility obtained from the "We/They are" formulations are consistent with those from the alternative constructions. When we fine-tuned the models using partisan tweets, there was a significant increase in ingroup solidarity and outgroup hostility, observed across all sentence starters. Conversely, when we fine-tuned the models on partisan tweets after removing positive "we" sentences and negative "they" sentences, the resulting models exhibited markedly lower levels of ingroup-outgroup bias, consistently across various sentence starters.

1.7. Study 1: Exploring specific identities

We tested if the ingroup solidarity and outgroup hostility effects with respect to four specific groups (women, men, US Republicans, US Democrats, Cambridge students). To do this, we prompted GPT-2-124M with "As X, we are" or "As X, they are"; for instance, for women we prompted with "As women, we are" for the ingroup sentences and "As women, they are" for the outgroup sentences. The resulting odds ratios are in line with the ones we observe with no group specified (see Supplementary Figure 11).

1.8. Study 1: Conversational prompt for base models

For base models, we also experimented with alternative prompts to ensure the robustness of our results to prompt variations and increase construct validity by using conversation-like prompts. As the base models are only capability of next token predictions, we came up with a prompt similar to the following:

Human: What kind of noises did dinosaurs make? Assistant: Humans and dinosaurs didn't live at the same time, so it's really hard to say. The best place to find out what noises dinosaurs made would be Human: yes they did Assistant: to guess, and that would probably require lots of reading and a certain amount of imagination, so we're not really prepared to do that. Human: you cant read Assistant: You canread? **Another Human: Hello everyone, I couldn't help overhearing your conversation. I'm in a bit of a creative challenge at the moment and could use your help. I need to complete this sentence - They are**

The bolded part is the actual prompt and the part before it provides context from conversations between humans and AI assistants in the HH-RLHF dataset [70]. We used the "chosen" portion of the dataset, which contains segments marked as helpful and harmless by human annotators. We then prompted a number of base models and conducted the same analysis as in Study 1 of the main manuscript. The results are illustrated in Supplementary Figure 13. We observed that although some models demonstrate reduced levels of ingroup solidarity relative to the default prompt, all models exhibit significant amounts of outgroup hostility that notably exceed the levels of ingroup solidarity. We hypothesize that this could be attributed to the presence of negative attitudes toward outgroups in the dialogues of the HH-RLHF dataset, as revealed by manual inspection. Interestingly, despite the majority of prompts displaying exclusively outgroup hostility,

some models still show substantial levels of ingroup solidarity. This suggests that while the ingroup-outgroup bias is influenced by context, it is more deeply entrenched than that.

1.9. Study 2: Partisan finetuning with different proportions of ingroup positive and outgroup negative sentences

See Supplementary Figure 14 for the effect of changing specific proportions of ingroup positive and outgroup negative sentences on the biases.

1.10. Study 3: Logistic regressions

We conducted a robustness check with non-mixed effects (ordinary) logistic regressions for Study 3 (with RoBERTa sentiment classification). The results (see Supplementary Table 26) are very similar to those of the mixed-effects logistic regressions presented in the results section.

2. Supplementary Figures

a

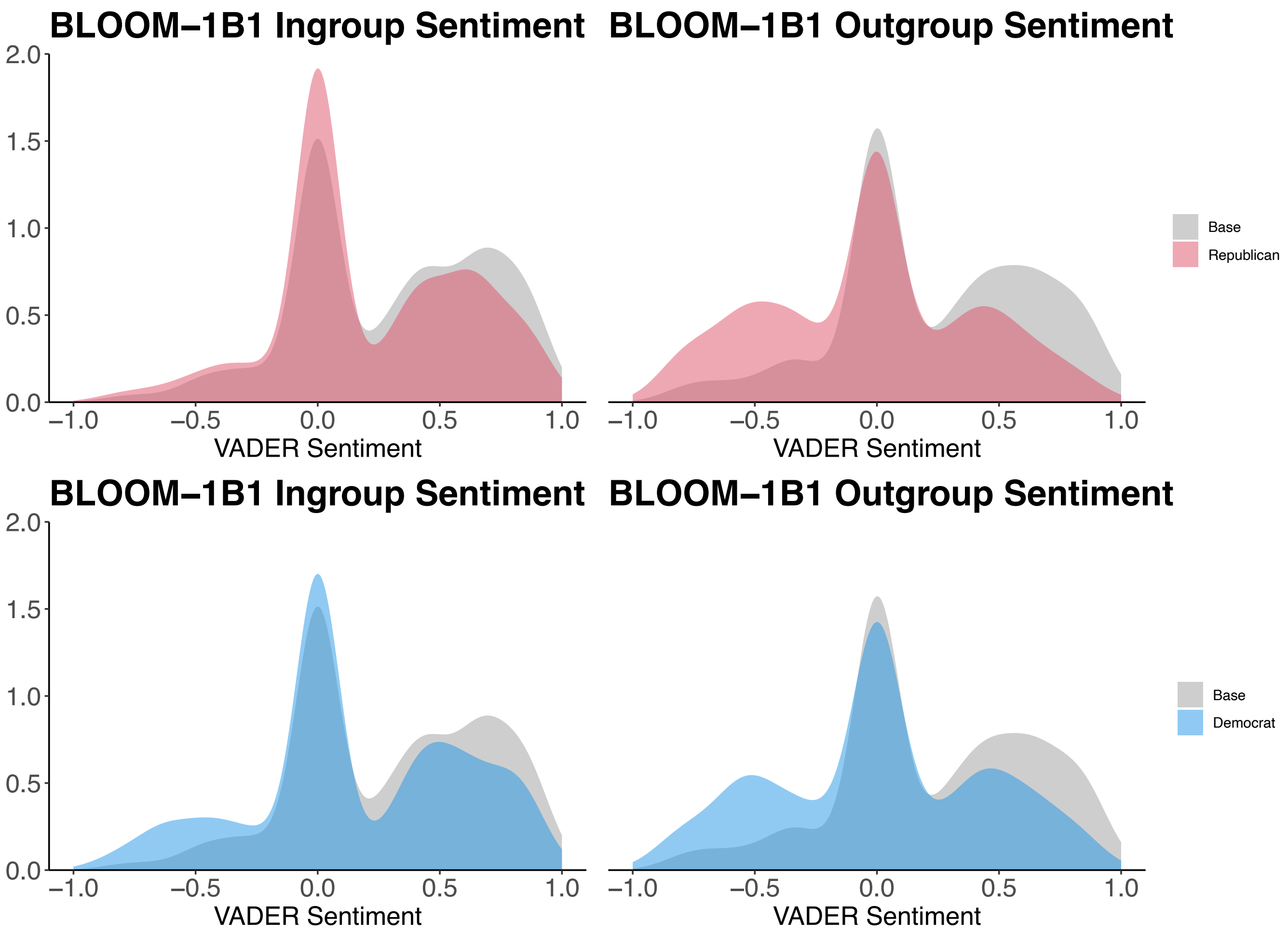

b

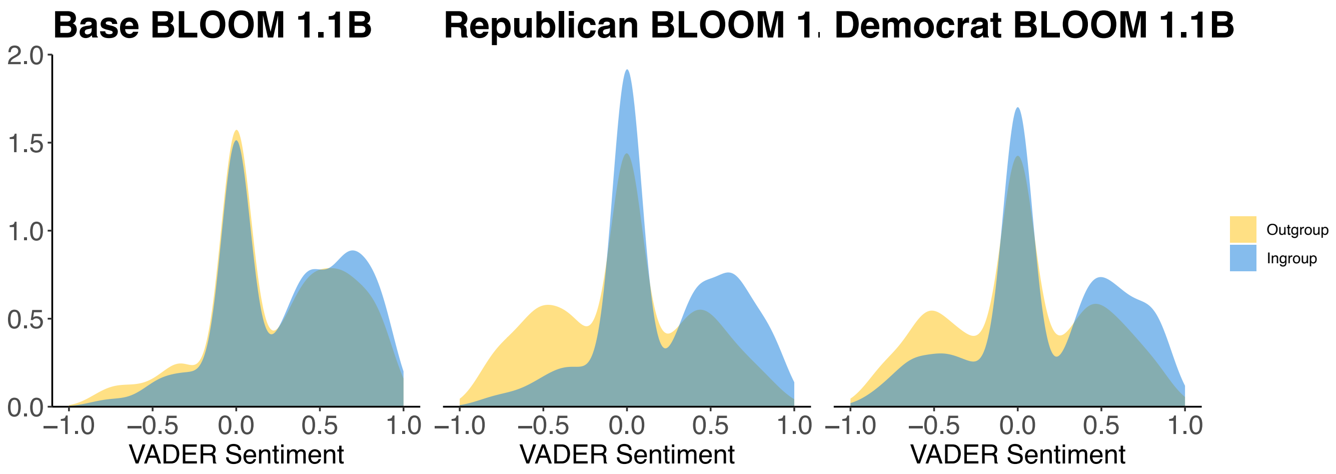

2.1. Supplementary Figure 1: Study 2: VADER sentiment scores for ingroup and outgroup sentences generated by BLOOM 1.1B, before and after fine-tuning on Republican or Democrat Twitter (now X) data.

a Results grouped by sentence type (ingroup/outgroup), N=6,000 sentences. **b** Results grouped by model version (pre/post fine-tuning). VADER scores range from -1 (most negative) to +1 (most positive), N=6,000 sentences.

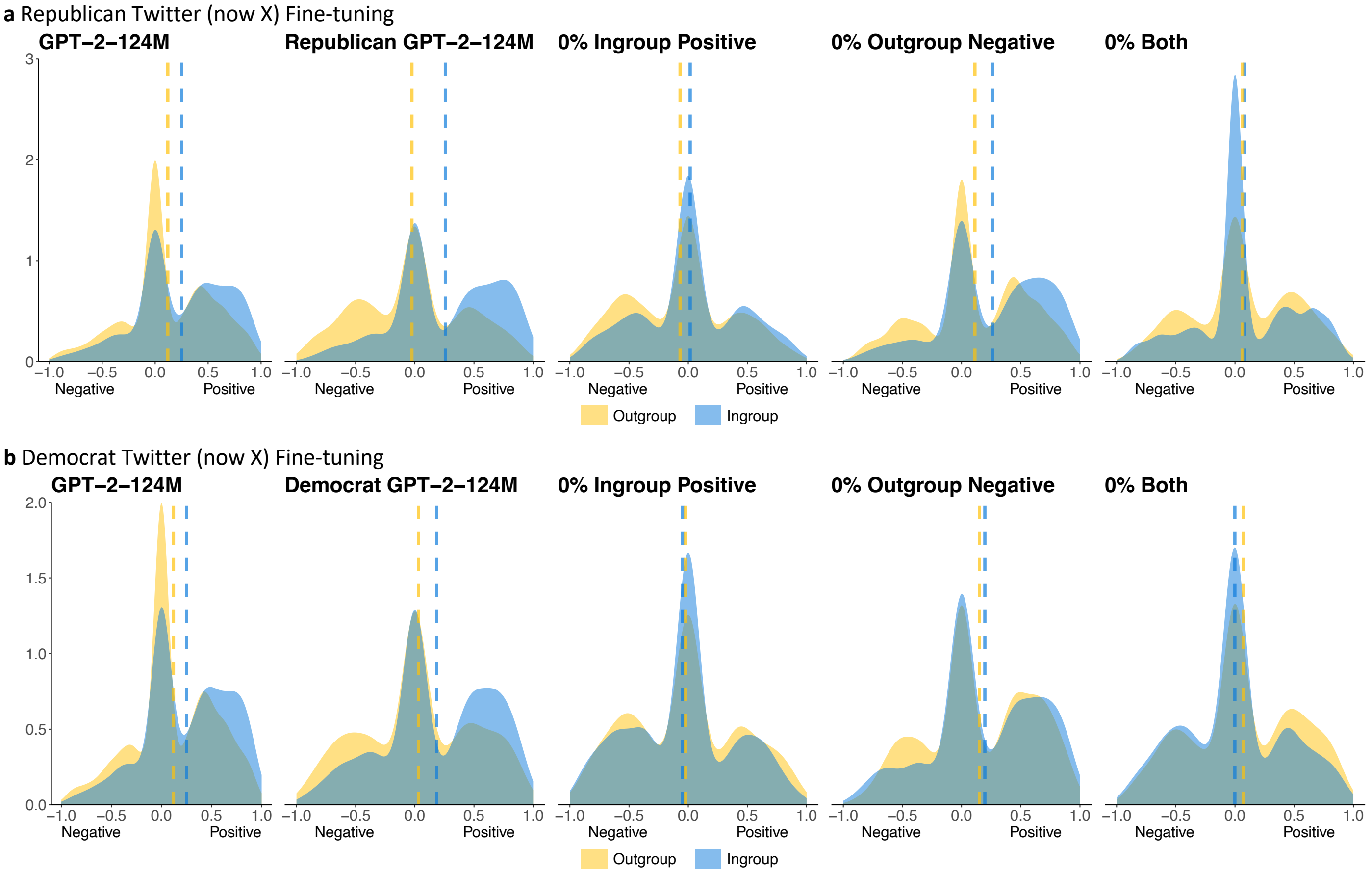

2.2. Supplementary Figure 2: Study 2: VADER sentiment score of model-generated sentences across fine-tuning settings.

VADER sentiment scores (x-axis; from -1 = Negative to +1 = Positive) for ingroup and outgroup sentences generated by GPT-2-124M and its variants fine-tuned on Republican and Democrat Twitter (now X) data, with selective removal of positive ingroup or negative outgroup training examples (N=20,000 sentences). See Methods for details.

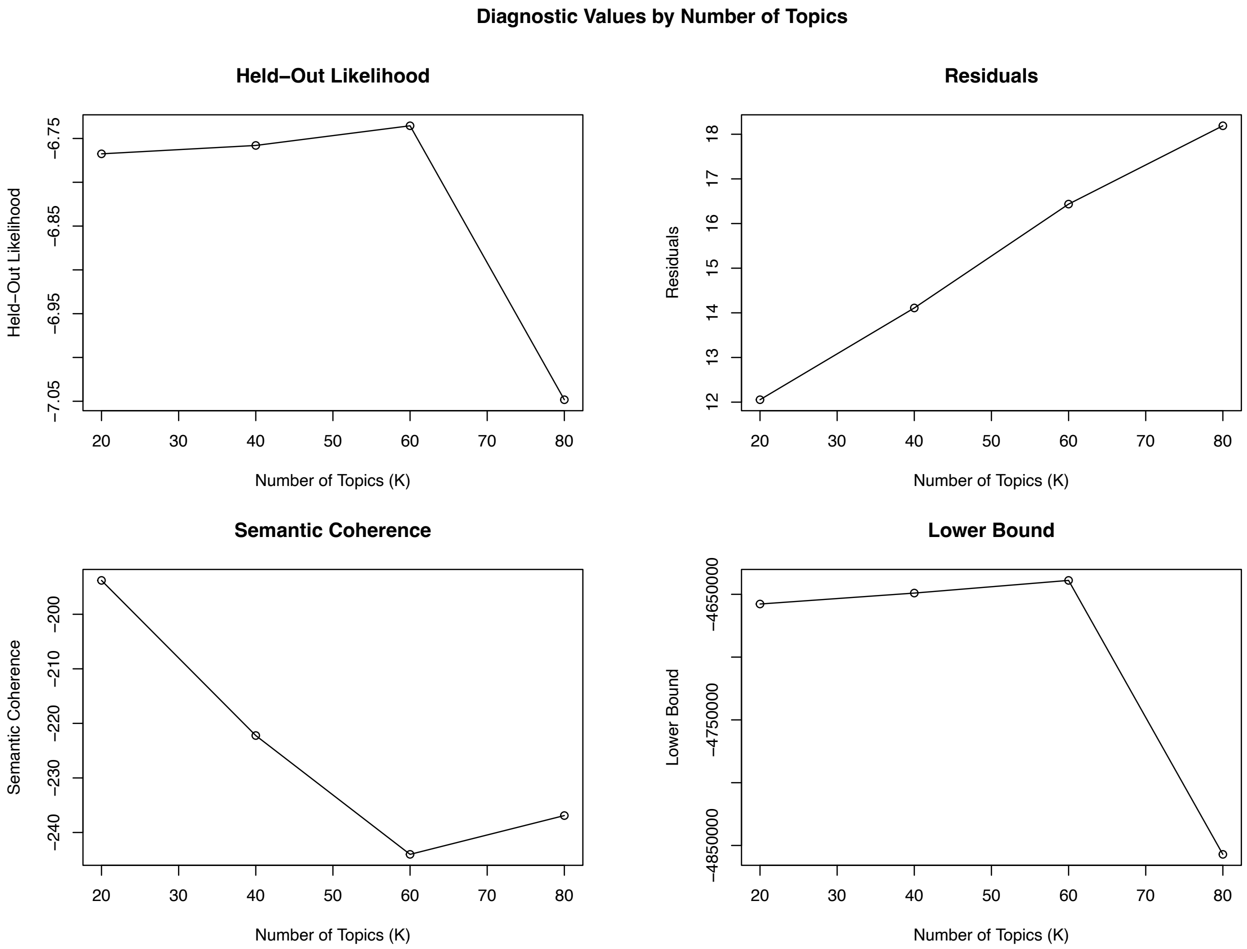

2.3. Supplementary Figure 3: Study 1: Diagnostic metrics for structural topic models across varying numbers of topics.

The plots show held-out likelihood (top left), residuals (top right), semantic coherence (bottom left), and lower bound values (bottom right) as functions of topic count (K). See Supplementary Section 4 for details.

a

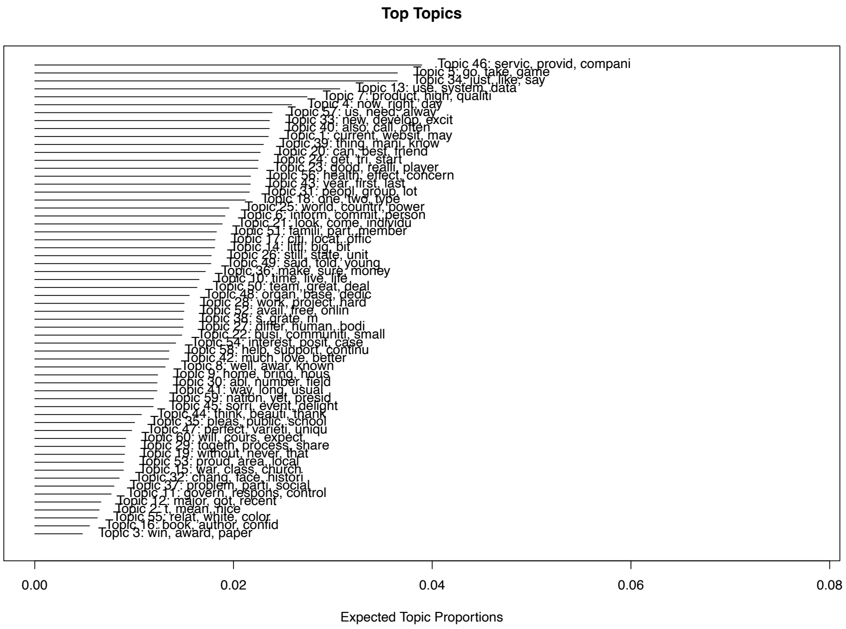

b

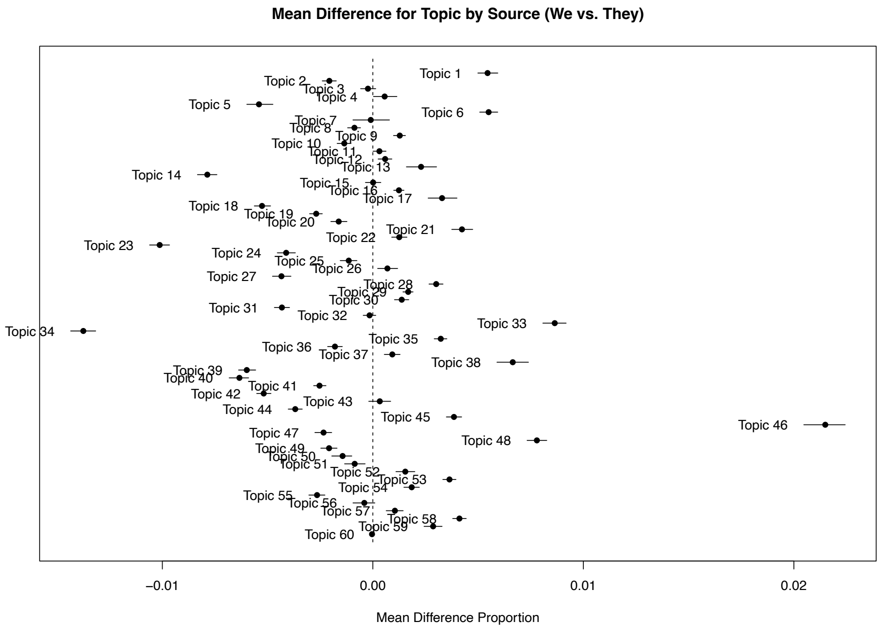

2.4. Supplementary Figure 4: Study 1: Structural topic modeling analysis of model-generated sentences.

a Expected topic proportions across the corpus generated by 51 models with the default promt, showing the relative frequency of different semantic themes. **b** Mean difference in topic proportions between “We are” and “They are” sentences, with positive values indicating topics more associated with “We are” statements (N=101,715 sentences). See Supplementary Section 4 for details.

a Ingroup solidarity

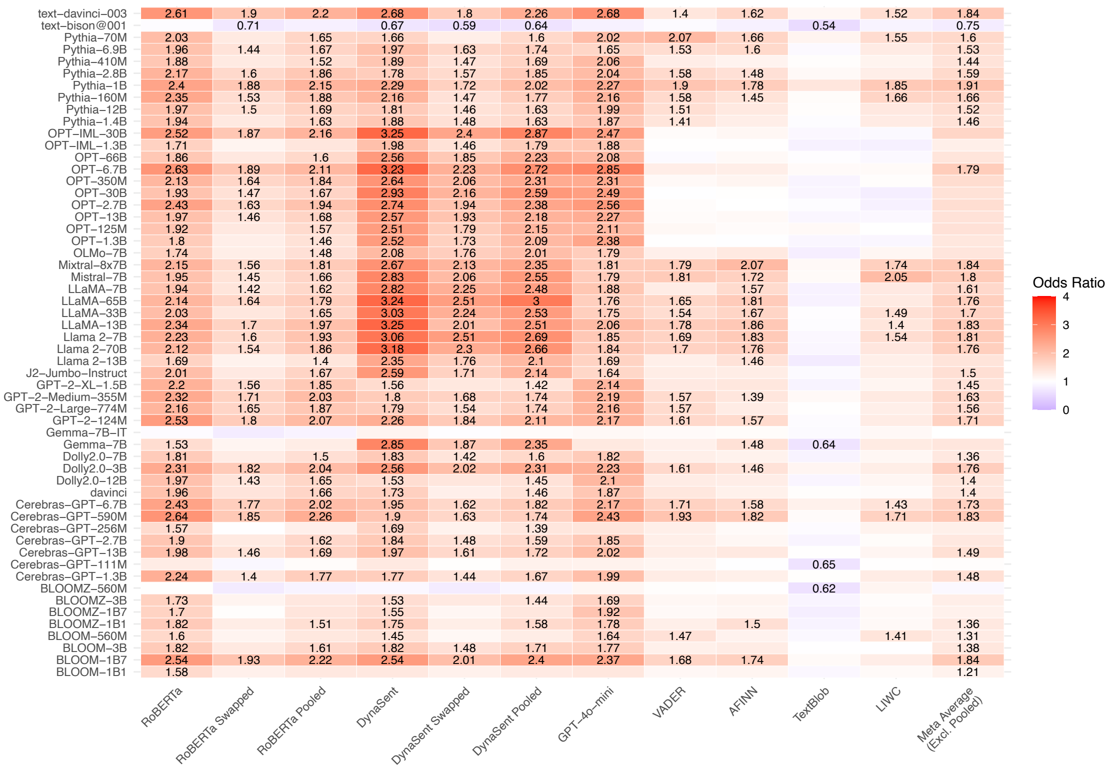

b Outgroup hostility

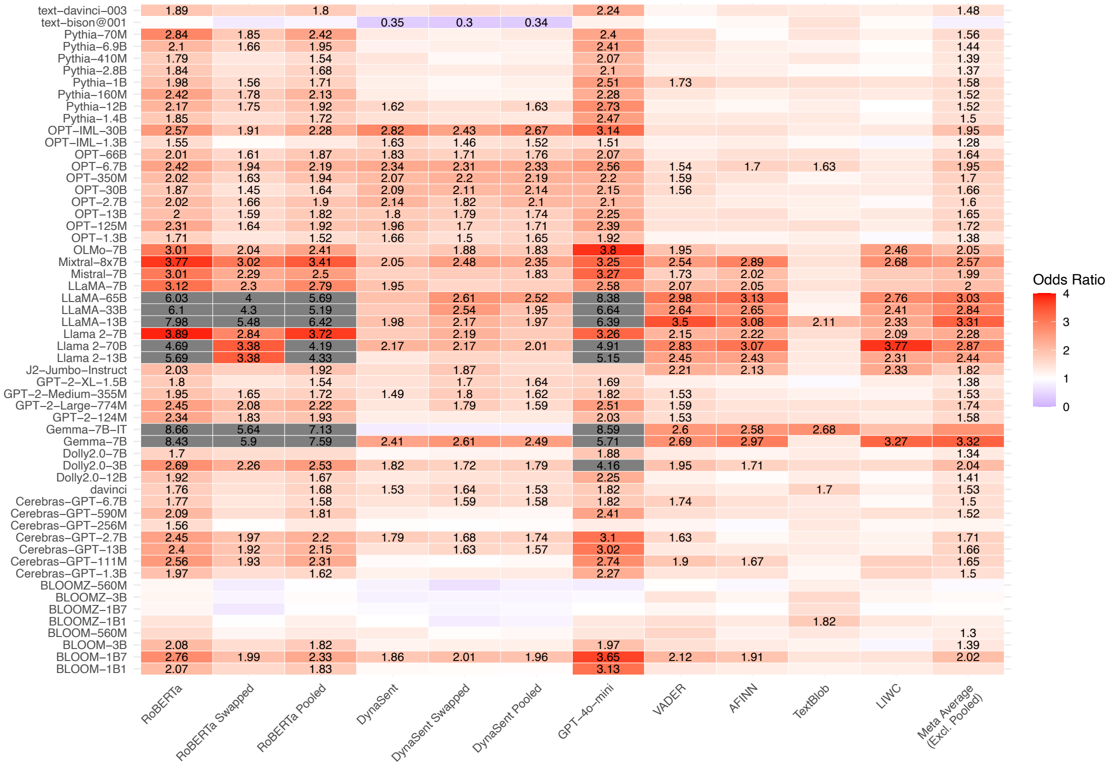

2.5. Supplementary Figure 5: Study 1: Logistic regression analysis across language models tested with the default prompt with different sentiment classifiers.

a Ingroup solidarity odds ratios. **b** Outgroup hostility odds ratios. N=112,000 sentences; 2,000 sentences per cell. Only statistically significant results (*p* < .0004, except for meta-analysis where p <.05) are shown. Cells in red indicate odds ratios > 1, representing higher ingroup solidarity or outgroup hostility. For example, an odds ratio of 2 indicates the ingroup sentence is twice as likely to be classified as positive compared to outgroup sentences. Odds ratios > 4 are depicted in grey. "Meta Average (Excl. Pooled)" refers to the average odds ratios across classifiers estimated using random-effects meta-analyses. See Supplementary Section 5 for details.

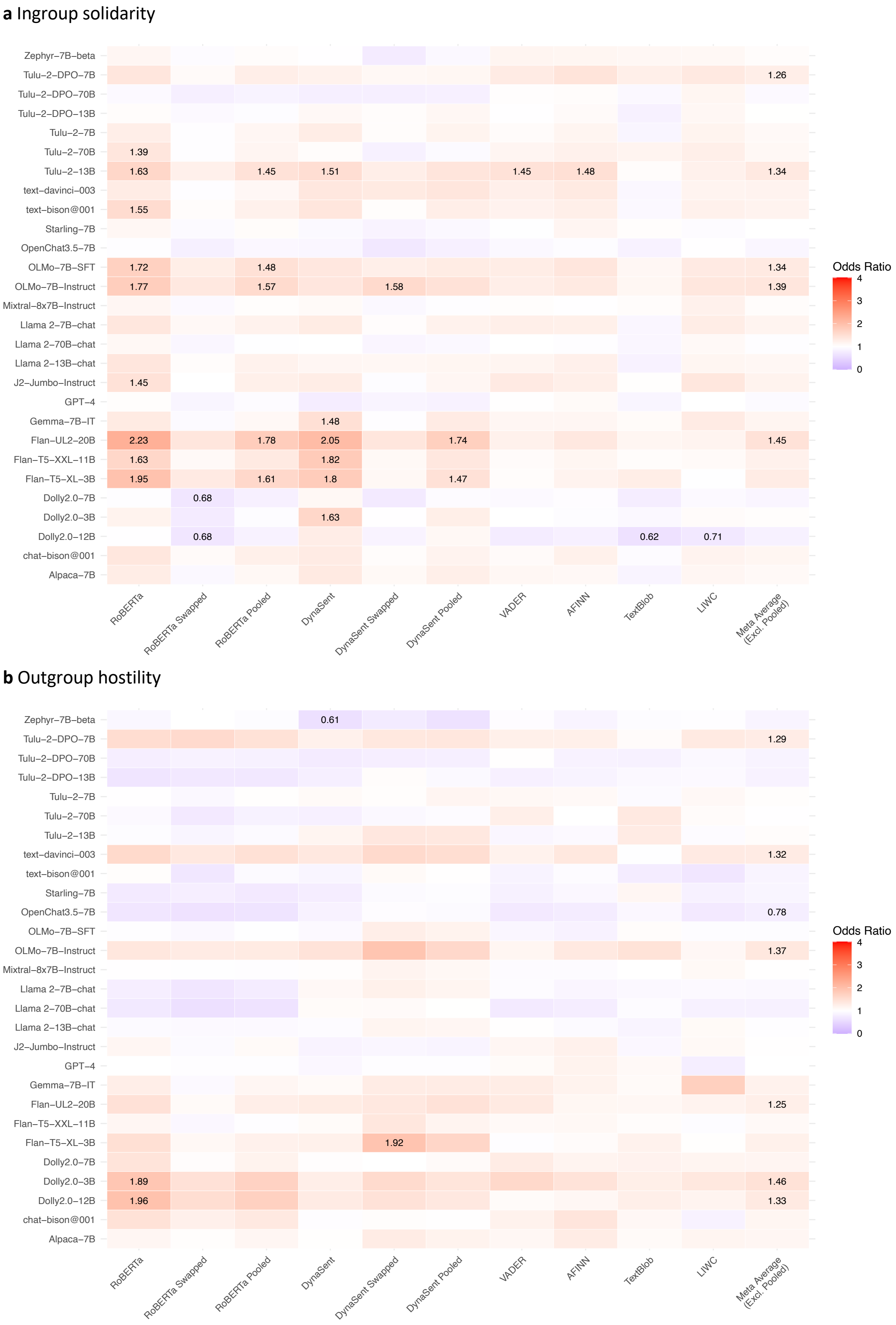

2.6. Supplementary Figure 6: Study 1: Logistic regression analysis across language models tested with the instruction prompt with different sentiment classifiers.

a Ingroup solidarity odds ratios. **b** Outgroup hostility odds ratios. N=76,000 sentences, 2,000 sentences per cell. Only statistically significant results (*p* < .0004, except for meta-analysis where p <.05) are shown. Cells in red indicate odds ratios > 1, representing higher ingroup solidarity or outgroup hostility. For example, an odds ratio of 2 indicates the ingroup sentence is twice as likely to be classified as positive compared to outgroup sentences. Odds ratios > 4 are depicted in grey. "Meta Average (Excl. Pooled)" refers to the average odds ratios across classifiers estimated using random-effects meta-analyses. See Supplementary Section 5 for details.

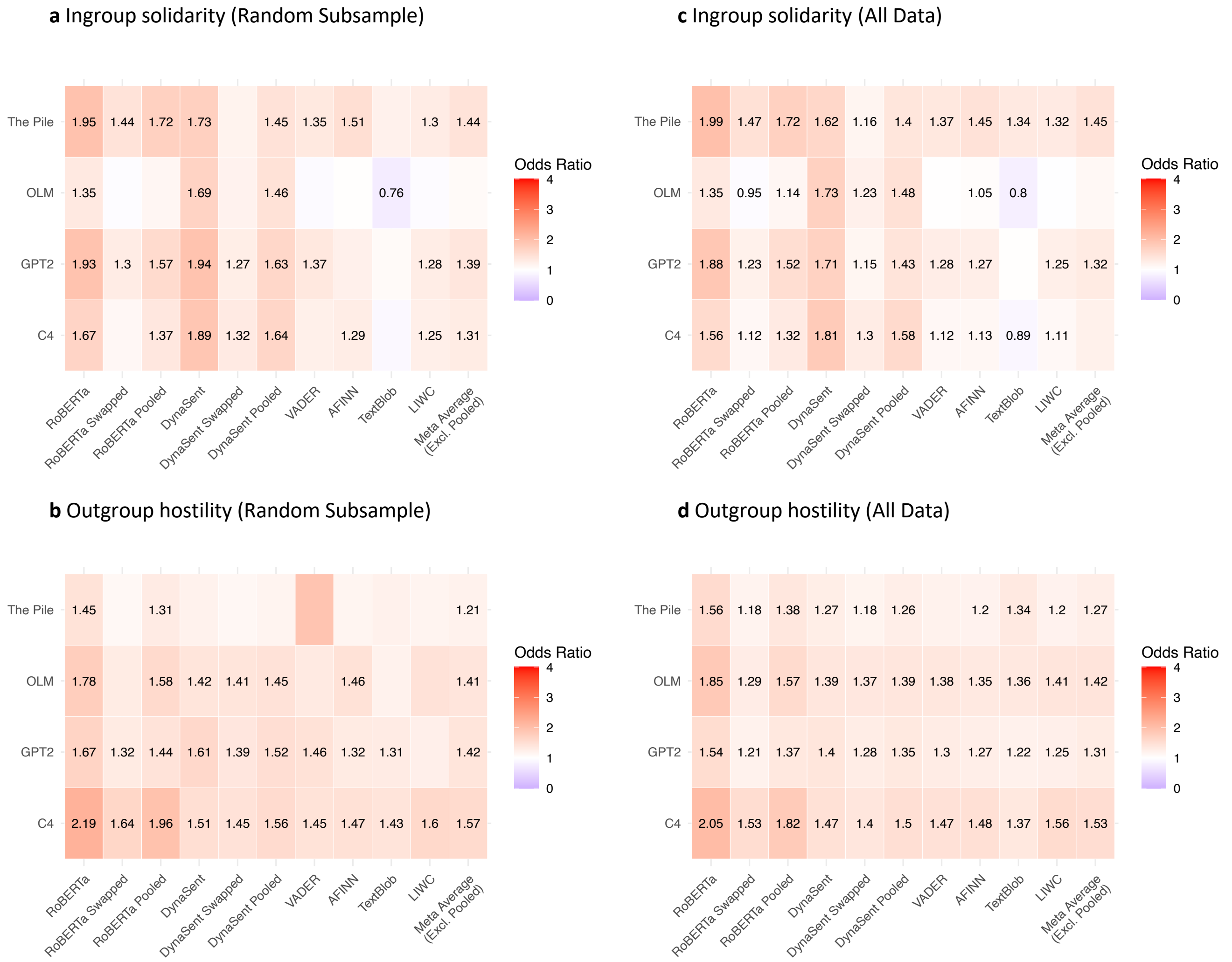

2.7. Supplementary Figure 7: Study 1: Logistic regression analysis across pertaining corpora with different sentiment classifiers.

a,**c** Ingroup solidarity odds ratios. **b**,**d** Outgroup hostility odds ratios. N=16,000 sentences for Random Subsample plots (a and b) and N=770,302 for All Data plots (c and d); 2,000 sentences per cell. Only statistically significant results ($p < .001$, except for meta-analysis where $p < .05$) are shown. Cells in red indicate odds ratios > 1, representing higher ingroup solidarity or outgroup hostility. For example, an odds ratio of 2 indicates the ingroup sentence is twice as likely to be classified as positive compared to outgroup sentences. “Meta Average (Excl. Pooled)” refers to the average odds ratios across classifiers estimated using random-effects meta-analyses. See Supplementary Section 5 for details.

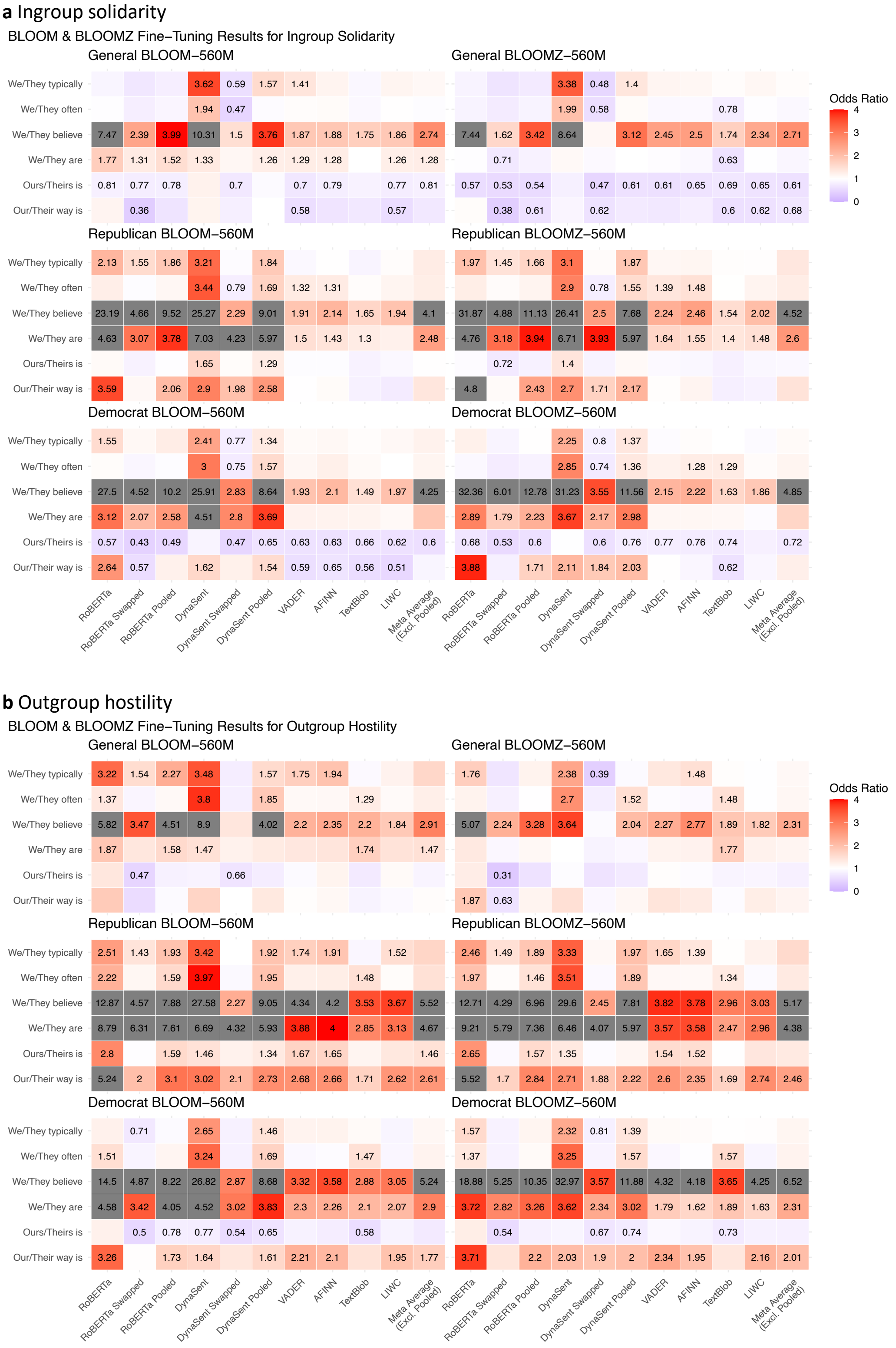

2.8. Supplementary Figure 8: Study 2: Logistic regression analysis for BLOOM-560M and BLOOMZ-560M with no or partisan fine-tuning and different prompting formulations and sentiment classifiers.

a Ingroup solidarity odds ratios. **b** Outgroup hostility odds ratios. N=72,000 sentences, 2,000 sentences per cell. Only statistically significant results (*p* < .001, except for meta-analysis where p <.05) are shown. Cells in red indicate odds ratios > 1, representing higher ingroup solidarity or outgroup hostility. For example, an odds ratio of 2 indicates the ingroup sentence is twice as likely to be classified as positive compared to outgroup sentences. Odds ratios > 4 are depicted in grey. “Meta Average (Excl. Pooled)” refers to the average odds ratios across classifiers estimated using random-effects meta-analyses. See Supplementary Section 6 for details.

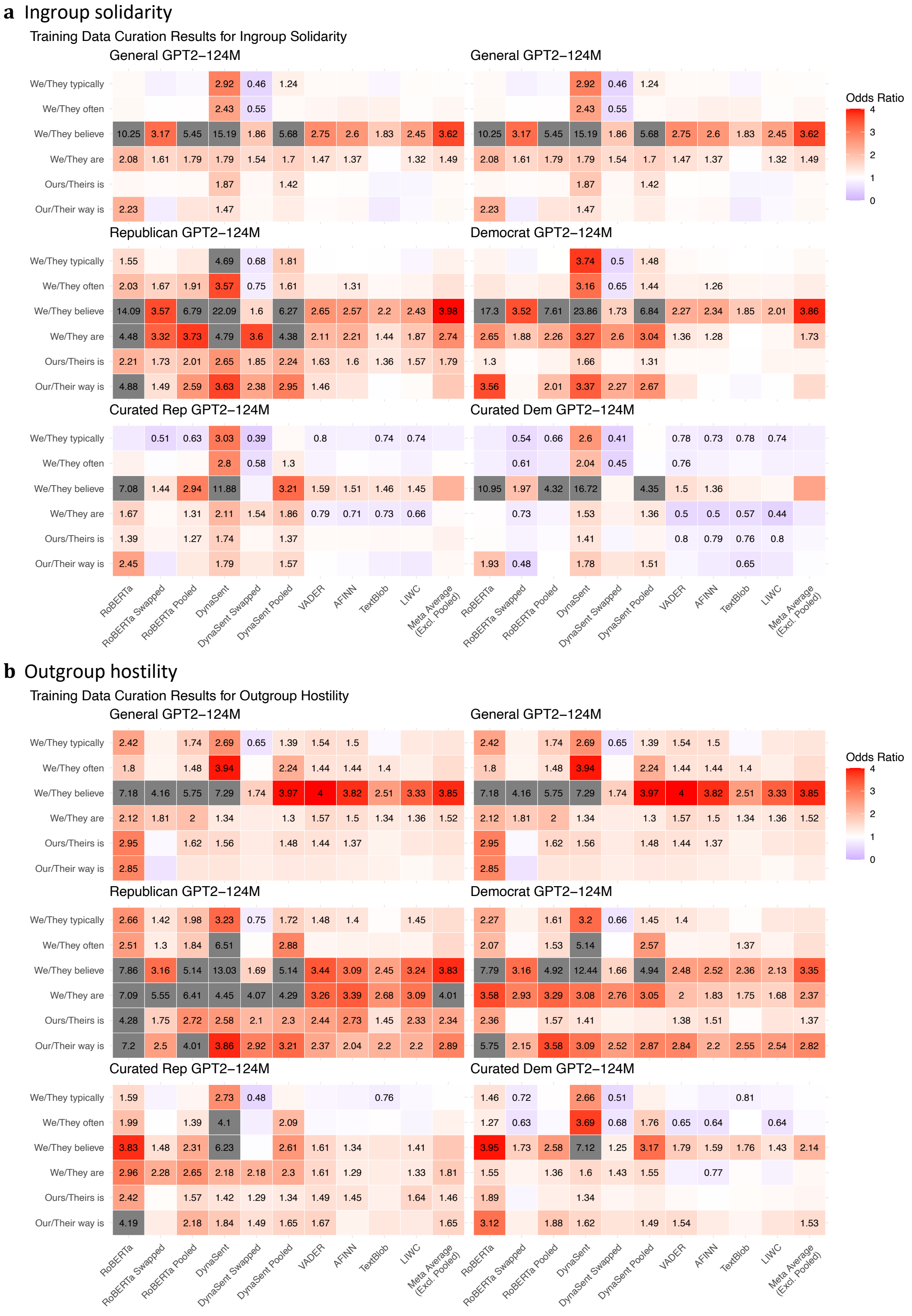

2.9. Supplementary Figure 9: Study 2: Logistic regression analysis for GPT2-124M with none, partisan, or curated (ingroup and outgroup sentences removed) fine-tuning and with different prompting formulations and sentiment classifiers.

a Ingroup solidarity odds ratios. **b** Outgroup hostility odds ratios. N=72,000 sentences, 2,000 sentences per cell. Only statistically significant results (p < .001, except for meta-analysis where p <.05) are shown. Cells in red indicate odds ratios > 1, representing higher ingroup solidarity or outgroup hostility. For example, an odds ratio of 2 indicates the ingroup sentence is twice as likely to be classified as positive compared to outgroup sentences. Odds ratios > 4 are depicted in grey. "Meta Average (Excl. Pooled)" refers to the average odds ratios across classifiers estimated using random-effects meta-analyses. See Supplementary Section 6 for details.

a Ingroup solidarity

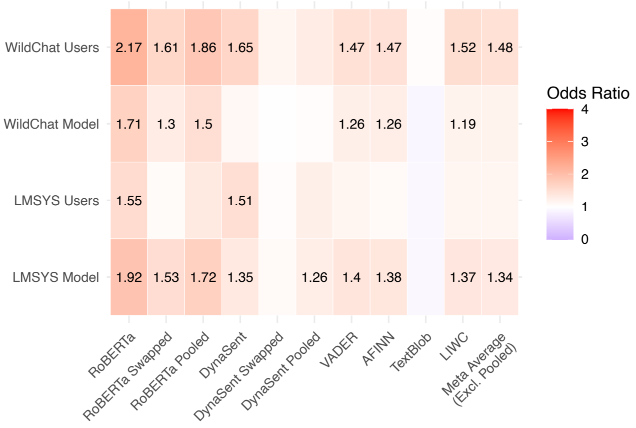

b Outgroup Hostility

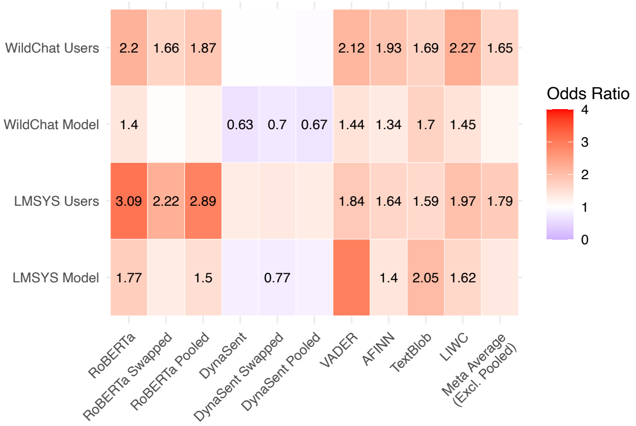

2.10. Supplementary Figure 10: Study 3: Logistic regression analysis of human-LLM conversations with different sentiment classifiers.

a Ingroup solidarity odds ratios. **b** Outgroup hostility odds ratios. N=25,395 sentences; 10,507 from WildChat Model, 2,453 from WildChat Users, 10,247 from LMSYS Models, and 2,188 from LMSYS Users. Only statistically significant results ($p < .001$, except for meta-analysis where $p < .05$) are shown. Cells in red indicate odds ratios > 1, representing higher ingroup solidarity or outgroup hostility. For example, an odds ratio of 2 indicates the ingroup sentence is twice as likely to be classified as positive compared to outgroup sentences. Odds ratios > 4 are depicted in grey. "Meta Average (Excl. Pooled)" refers to the average odds ratios across classifiers estimated using random-effects meta-analyses. See Supplementary Section 5 for details.

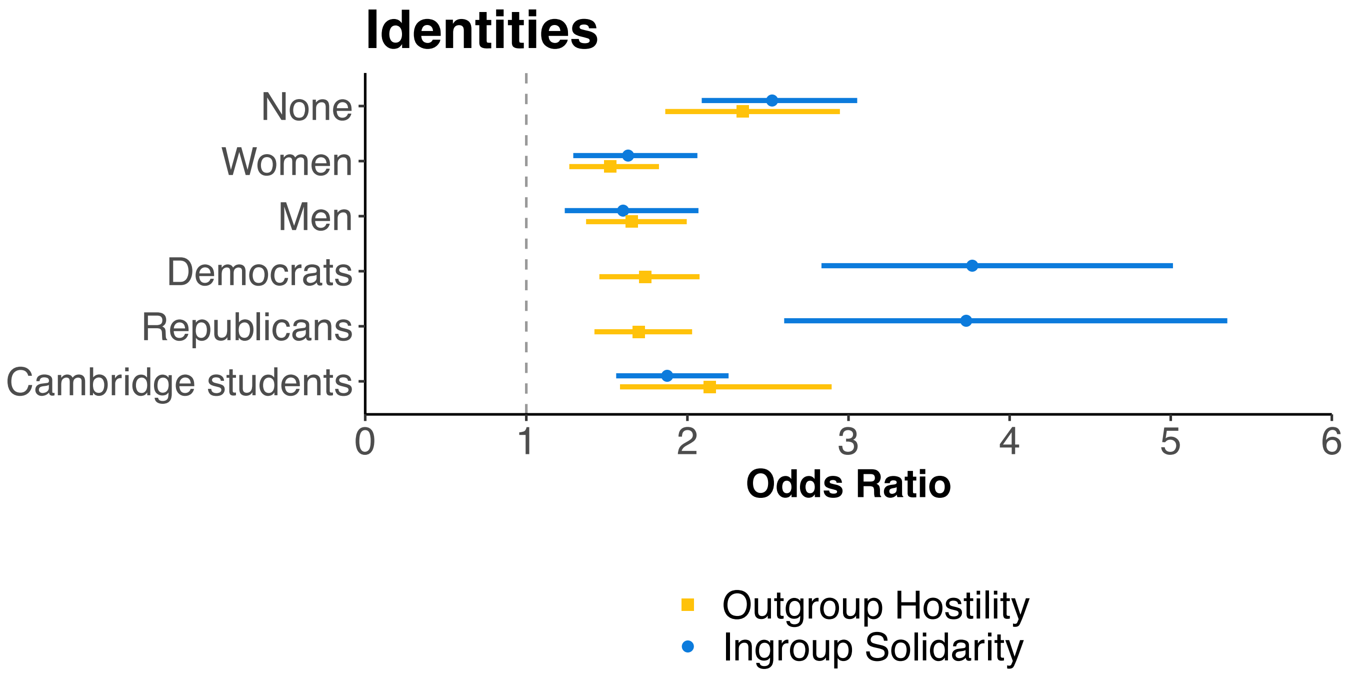

2.11. Supplementary Figure 11: Study 1: Logistic regression analysis of GPT-2-124M when mentioning specific group identities.

We prompted GPT-2-124M to generate ingroup and outgroup sentences based on specific identities (for instance, women) and no specific group identity (the usual setting in the rest of our experiment). For example, we prompted the GPT-2-124M with "As women, we are" to generate the ingroup sentences and "As women, they are" for the outgroup. The plots show the results of individual logistic regressions fit on 2,000 sentences from a specific LLM predicting positive (or negative) sentiment based on whether a sentence is ingroup (or outgroup), controlling for the number of words and type-to-token ratio (N=12,000 sentences). Data are presented as odds ratios with error bars for 95% confidence intervals. See Supplementary Section 7 for details.

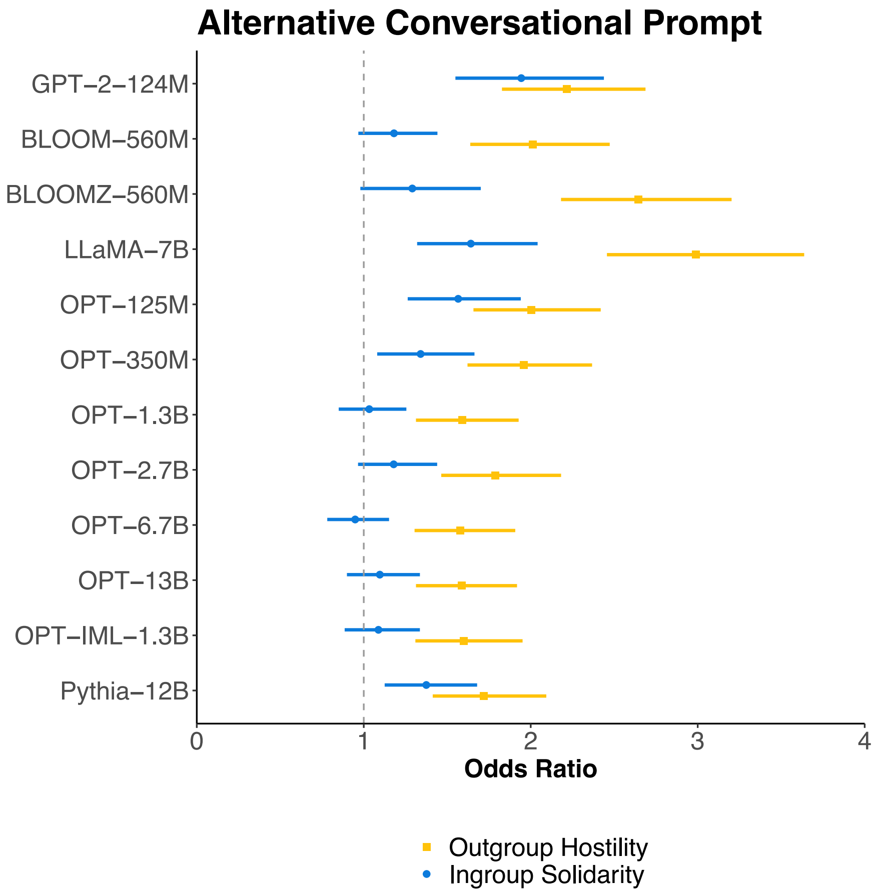

2.12. Supplementary Figure 12: Study 1: Logistic regression analysis of language models tested with an alternative conversation-like prompt.

The plots show the results of individual logistic regressions fit on 2,000 sentences from a specific LLM predicting positive (or negative) sentiment based on whether a sentence is ingroup (or outgroup), controlling for the number of words and type-to-token ratio (N=24,000 sentences). Data are presented as odds ratios with error bars for 95% confidence intervals. See Supplementary Section 8 for details.

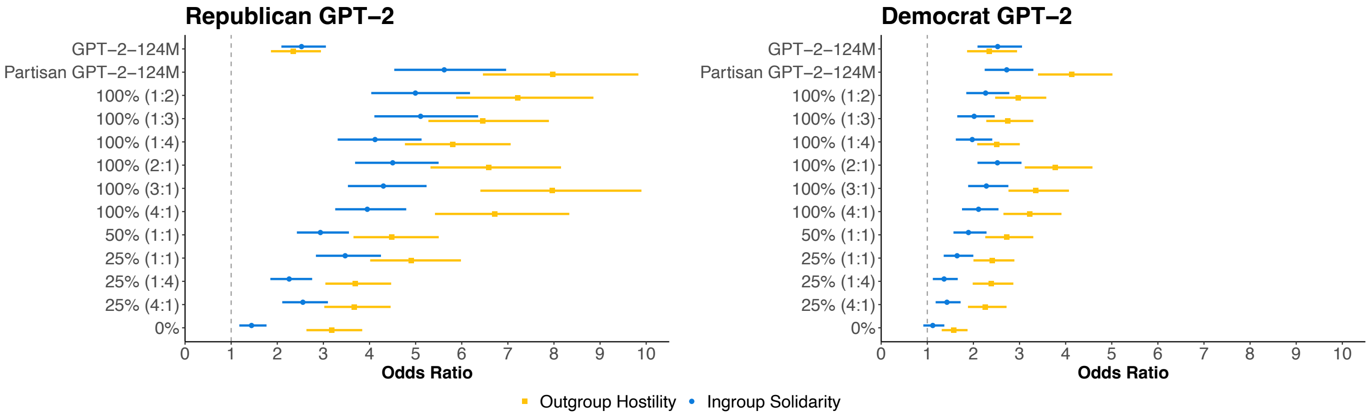

2.13. Supplementary Figure 13: Study 2: Logistic regression analysis of the effects of different training data compositions on finetuning outcomes.

The plots show the results of individual logistic regressions fit on 2,000 sentences from a specific finetuned LLM predicting positive (or negative) sentiment based on whether a sentence is ingroup (or outgroup), controlling for the number of words and type-to-token ratio (N=52,000 sentences). Each row represents results of an LLM finetuned with a different training data sampling strategy. The notation "X% (Y:Z)" indicates that X% of the original ingroup positive and outgroup negative sentences is retained, with a Y:Z ratio between ingroup positive and outgroup negative sentences. For example, "25% (1:4)" means the total data is reduced to 25% of the original size, with ingroup positive sentences being 1/4 as numerous as outgroup negative sentences. "0%" indicates that all ingroup positive and outgroup negative sentences have been removed before fine-tuning. Data are presented as odds ratios with error bars for 95% confidence intervals. See Supplementary Section 9 for details.

3. Supplementary Tables

3.1. Supplementary Table 1: Study 1: Ratio of sentences kept post filtering (default prompt).

Ratio of sentences kept after quality filtering described in Supplementary Section 2 for 51 LLMs tested with the default prompt for which data was collected before September 2023.

Model	% Good Sentence (We)	% Good Sentence (They)
BLOOM-1B1	35.84189	47.63066
BLOOM-1B7	35.80927	41.92283
BLOOM-3B	33.79784	44.87732
BLOOM-560M	37.27511	52.94227
BLOOMZ-1B1	35.06366	47.31352
BLOOMZ-1B7	35.98343	49.73124
BLOOMZ-3B	37.06725	48.20288
BLOOMZ-560M	37.86466	53.35323
Cerebras-GPT-1.3B	35.17987	44.24836
Cerebras-GPT-2.7B	35.05258	46.81686
Cerebras-GPT-6.7B	35.52036	44.87952
Cerebras-GPT-13B	32.93599	44.31911
Cerebras-GPT-111M	33.51308	43.71180
Cerebras-GPT-256M	33.83225	38.24923
Cerebras-GPT-590M	34.19168	44.96112
Dolly2.0-3B	26.85944	40.70248
Dolly2.0-7B	30.54299	44.69344
Dolly2.0-12B	31.75398	45.93918
GPT-2-124M	40.37771	55.53623
GPT-2-Large-774M	38.32347	51.69243
GPT-2-Medium-355M	40.79678	56.62109
GPT-2-XL-1.5B	38.32853	52.00079
J2-Jumbo-Instruct	8.24708	40.63225
LLaMA-7B	31.45870	52.34554
LLaMA-13B	31.01427	53.76202
LLaMA-33B	31.11718	51.56279
LLaMA-65B	30.43222	49.79095
Llama 2-7B	29.56539	54.27316
Llama 2-13B	31.65468	54.04595
Llama 2-70B	30.82000	54.94000
OPT-1.3B	36.08230	48.13764
OPT-2.7B	36.78924	47.60135
OPT-6.7B	34.44109	46.95296
OPT-13B	34.21719	47.38253
OPT-30B	35.46331	47.86950
OPT-66B	34.57411	47.09156
OPT-125M	41.32875	51.00708
OPT-350M	39.70508	49.59024
OPT-IML-1.3B	34.48446	48.03593
OPT-IML-30B	34.03788	46.23257
Pythia-1.4B	30.88540	38.71630
Pythia-1B	30.79407	39.69094
Pythia-2.8B	30.52154	38.84142
Pythia-6.9B	31.88493	42.89527
Pythia-12B	31.37255	40.61988
Pythia-70M	33.91139	40.54834
Pythia-160M	40.35311	44.33802
Pythia-410M	28.50035	37.81067
davinci	75.80000	81.04000
text-bison@001	26.46000	30.56000
text-davinci-003	58.14477	67.91344

3.2. Supplementary Table 2: Study 1: Ratio of sentences kept after filtering (instruction prompt).

Ratio of sentences kept after quality filtering described in Supplementary Section 2 for 15 LLMs tested with the instruction prompt for which data was collected before September 2023.

Model	% Good Sentence (We)	% Good Sentence (They)
Alpaca-7B	29.15556	58.66667
Dolly2.0-3B	60.38121	68.01661
Dolly2.0-7B	64.37288	72.30823
Dolly2.0-12B	54.24016	67.17896
Flan-T5-XL-3B	59.16667	65.46667
Flan-T5-XXL-11B	67.23333	76.23333
Flan-UL2-20B	59.13333	59.36667
GPT-4	61.60000	84.43333
J2-Jumbo-Instruct	56.06667	71.90000
Llama 2-7B-chat	34.44481	64.70981
Llama 2-13B-chat	37.33333	71.35712
Llama 2-70B-chat	28.58732	57.89942
chat-bison@001	57.70000	76.46667
text-bison@001	60.03367	79.73746
text-davinci-003	55.86667	78.73333

3.3. Supplementary Table 3: Study 1: Ingroup solidarity and outgroup hostility of LLMs tested with the default prompt (Part 1).

Each value represents an odds ratio from a separate logistic regression fitted on a total of 2,000 ingroup and outgroup sentences from a specific LLM predicting whether the sentence is positive (for ingroup solidarity) based on whether the sentence is ingroup (vs. outgroup), and negative (for outgroup hostility) based on whether the sentence is outgroup (vs. ingroup), and control variables. Tests are two-sided, and p-values are not adjusted for multiple comparisons. We consider the results statistically significant if p <.0004, obtained by dividing .05 by the total number of tests with the default prompt (112). Sentence sentiment determined using RoBERTa. See Methods for more details. Data are presented as OR (z value, p value, 95% CI).

	Ingroup Solidarity	Outgroup Hostility
GPT-2-124M	2.5259 (9.5648, p=0.0000, [2.0891, 3.0541])	2.3426 (7.2767, p=0.0000, [1.8626, 2.9463])
GPT-2-Medium-355M	2.3156 (8.6639, p=0.0000, [1.9150, 2.8000])	1.9455 (5.8661, p=0.0000, [1.5576, 2.4300])
GPT-2-Large-774M	2.1572 (8.0283, p=0.0000, [1.7881, 2.6026])	2.4531 (7.6886, p=0.0000, [1.9515, 3.0836])
GPT-2-XL-1.5B	2.2033 (8.1656, p=0.0000, [1.8227, 2.6633])	1.8026 (5.1603, p=0.0000, [1.4411, 2.2547])
davinci	1.9617 (6.6275, p=0.0000, [1.6073, 2.3943])	1.7627 (5.0636, p=0.0000, [1.4154, 2.1952])
text-davinci-003	2.6132 (10.0602, p=0.0000, [2.1672, 3.1510])	1.8891 (4.4775, p=0.0000, [1.4300, 2.4957])
BLOOM-560M	1.5990 (5.1445, p=0.0000, [1.3372, 1.9121])	1.5763 (3.1016, p=0.0019, [1.1823, 2.1015])
BLOOM-1B1	1.5792 (5.0028, p=0.0000, [1.3204, 1.8888])	2.0670 (5.0556, p=0.0000, [1.5599, 2.7389])
BLOOM-1B7	2.5394 (9.7903, p=0.0000, [2.1072, 3.0602])	2.7550 (6.7809, p=0.0000, [2.0555, 3.6927])
BLOOM-3B	1.8172 (6.4118, p=0.0000, [1.5139, 2.1812])	2.0774 (5.3739, p=0.0000, [1.5912, 2.7123])
BLOOMZ-560M	1.0209 (0.2187, p=0.8269, [0.8483, 1.2286])	1.1262 (0.7787, p=0.4361, [0.8351, 1.5187])
BLOOMZ-1B1	1.8232 (6.4153, p=0.0000, [1.5175, 2.1903])	1.4238 (2.3581, p=0.0184, [1.0615, 1.9098])
BLOOMZ-1B7	1.6963 (5.2438, p=0.0000, [1.3923, 2.0668])	1.1341 (0.8923, p=0.3722, [0.8602, 1.4951])
BLOOMZ-3B	1.7285 (5.7430, p=0.0000, [1.4340, 2.0835])	1.1479 (0.9929, p=0.3208, [0.8743, 1.5072])
OPT-125M	1.9158 (6.8512, p=0.0000, [1.5907, 2.3074])	2.3139 (7.6581, p=0.0000, [1.8668, 2.8681])
OPT-350M	2.1260 (7.7243, p=0.0000, [1.7557, 2.5745])	2.0234 (6.7907, p=0.0000, [1.6510, 2.4799])
OPT-1.3B	1.8024 (6.0957, p=0.0000, [1.4914, 2.1783])	1.7122 (5.0886, p=0.0000, [1.3919, 2.1063])
OPT-2.7B	2.4327 (9.0031, p=0.0000, [2.0046, 2.9521])	2.0242 (6.9314, p=0.0000, [1.6583, 2.4710])
OPT-6.7B	2.6329 (9.8253, p=0.0000, [2.1705, 3.1937])	2.4220 (8.4262, p=0.0000, [1.9716, 2.9754])
OPT-13B	1.9743 (6.9265, p=0.0000, [1.6286, 2.3933])	1.9993 (6.7390, p=0.0000, [1.6344, 2.4456])
OPT-30B	1.9331 (6.6477, p=0.0000, [1.5917, 2.3477])	1.8680 (6.1417, p=0.0000, [1.5303, 2.2802])
OPT-66B	1.8617 (6.3029, p=0.0000, [1.5346, 2.2587])	2.0056 (6.8497, p=0.0000, [1.6434, 2.4475])
OPT-IML-1.3B	1.7069 (5.3324, p=0.0000, [1.4024, 2.0776])	1.5492 (4.4662, p=0.0000, [1.2784, 1.8773])
OPT-IML-30B	2.5186 (9.1190, p=0.0000, [2.0651, 3.0717])	2.5744 (9.0893, p=0.0000, [2.0995, 3.1567])

3.4. Supplementary Table 4: Study 1: Ingroup solidarity and outgroup hostility of LLMs tested with the default prompt (Part 2).

Each value represents an odds ratio from a separate logistic regression fitted on a total of 2,000 ingroup and outgroup sentences from a specific LLM predicting whether the sentence is positive (for ingroup solidarity) based on whether the sentence is ingroup (vs. outgroup), and negative (for outgroup hostility) based on whether the sentence is outgroup (vs. ingroup), and control variables. Tests are two-sided, and p-values are not adjusted for multiple comparisons. We consider the results statistically significant if p <.0004, obtained by dividing .05 by the total number of tests with the default prompt (112). Sentence sentiment determined using RoBERTa. See Methods for more details. Data are presented as OR (z value, p value, 95% CI).

	Ingroup Solidarity	Outgroup Hostility
Pythia-70M	2.0326 (6.4003, p=0.0000, [1.6358, 2.5257])	2.8448 (8.1764, p=0.0000, [2.2142, 3.6550])
Pythia-160M	2.3510 (8.3732, p=0.0000, [1.9247, 2.8719])	2.4165 (7.3506, p=0.0000, [1.9099, 3.0575])
Pythia-410M	1.8807 (6.6457, p=0.0000, [1.5610, 2.2657])	1.7869 (4.8909, p=0.0000, [1.4160, 2.2549])
Pythia-1B	2.4049 (9.0417, p=0.0000, [1.9883, 2.9087])	1.9762 (5.7000, p=0.0000, [1.5635, 2.4977])
Pythia-1.4B	1.9402 (7.0313, p=0.0000, [1.6129, 2.3340])	1.8494 (5.0617, p=0.0000, [1.4576, 2.3465])
Pythia-2.8B	2.1692 (7.9754, p=0.0000, [1.7933, 2.6239])	1.8391 (5.2161, p=0.0000, [1.4628, 2.3122])
Pythia-6.9B	1.9578 (7.0556, p=0.0000, [1.6245, 2.3595])	2.1026 (5.7790, p=0.0000, [1.6342, 2.7053])
Pythia-12B	1.9702 (6.9138, p=0.0000, [1.6256, 2.3878])	2.1681 (6.5591, p=0.0000, [1.7205, 2.7321])
Dolly2.0-3B	2.3146 (8.6663, p=0.0000, [1.9145, 2.7984])	2.6890 (8.2503, p=0.0000, [2.1259, 3.4013])
Dolly2.0-7B	1.8074 (6.2162, p=0.0000, [1.4997, 2.1782])	1.7028 (4.1774, p=0.0000, [1.3265, 2.1859])
Dolly2.0-12B	1.9666 (7.1316, p=0.0000, [1.6330, 2.3683])	1.9191 (5.2695, p=0.0000, [1.5059, 2.4456])
Cerebras-GPT-111M	1.2949 (2.5983, p=0.0094, [1.0656, 1.5736])	2.5610 (7.3023, p=0.0000, [1.9897, 3.2963])
Cerebras-GPT-256M	1.5668 (4.1743, p=0.0000, [1.2690, 1.9345])	1.5562 (3.7701, p=0.0002, [1.2366, 1.9585])
Cerebras-GPT-590M	2.6415 (10.1871, p=0.0000, [2.1912, 3.1843])	2.0893 (5.5579, p=0.0000, [1.6112, 2.7093])
Cerebras-GPT-1.3B	2.2418 (8.5181, p=0.0000, [1.8618, 2.6994])	1.9658 (5.4592, p=0.0000, [1.5423, 2.5058])
Cerebras-GPT-2.7B	1.8951 (6.7031, p=0.0000, [1.5720, 2.2846])	2.4486 (7.4500, p=0.0000, [1.9346, 3.0990])
Cerebras-GPT-6.7B	2.4322 (9.0635, p=0.0000, [2.0069, 2.9476])	1.7705 (4.7128, p=0.0000, [1.3961, 2.2454])
Cerebras-GPT-13B	1.9789 (7.2317, p=0.0000, [1.6447, 2.3810])	2.3953 (7.0384, p=0.0000, [1.8781, 3.0549])
Mistral-7B	1.9475 (7.1524, p=0.0000, [1.6224, 2.3378])	3.0103 (6.7150, p=0.0000, [2.1823, 4.1524])
Mixtral-8x7B	2.1475 (8.1493, p=0.0000, [1.7869, 2.5808])	3.7656 (8.0650, p=0.0000, [2.7283, 5.1973])
J2-Jumbo-Instruct	2.0091 (7.5709, p=0.0000, [1.6771, 2.4068])	2.0298 (4.5397, p=0.0000, [1.4952, 2.7554])
OLMo-7B	1.7432 (6.0294, p=0.0000, [1.4551, 2.0884])	3.0147 (6.4760, p=0.0000, [2.1588, 4.2101])
text-bison@001	0.9625 (-0.4262, p=0.6700, [0.8072, 1.1476])	1.0335 (0.2166, p=0.8286, [0.7667, 1.3933])

3.5. Supplementary Table 5: Study 1: Ingroup solidarity and outgroup hostility of outlier LLMs tested with the default prompt.

Each value represents an odds ratio from a separate logistic regression fitted on a total of 2,000 ingroup and outgroup sentences from a specific LLM predicting whether the sentence is positive (for ingroup solidarity) based on whether the sentence is ingroup (vs. outgroup), and negative (for outgroup hostility) based on whether the sentence is outgroup (vs. ingroup), and control variables. Tests are two-sided, and p-values are not adjusted for multiple comparisons. We consider the results statistically significant if $p < .0004$, obtained by dividing .05 by the total number of tests with the default prompt (112). Sentence sentiment determined using RoBERTa. See Methods for more details. Data are presented as OR (z value, p value, 95% CI).

	Ingroup Solidarity	Outgroup Hostility
LLaMA-7B	1.9366 (7.0993, p=0.0000, [1.6136, 2.3243])	3.1227 (5.8024, p=0.0000, [2.1256, 4.5876])
LLaMA-13B	2.3378 (9.0103, p=0.0000, [1.9435, 2.8121])	7.9784 (8.4427, p=0.0000, [4.9265, 12.9210])
LLaMA-33B	2.0252 (7.5950, p=0.0000, [1.6881, 2.4298])	6.0970 (7.6198, p=0.0000, [3.8297, 9.7066])
LLaMA-65B	2.1429 (8.2176, p=0.0000, [1.7867, 2.5700])	6.0287 (7.4037, p=0.0000, [3.7470, 9.7000])
Llama 2-7B	2.2291 (8.5214, p=0.0000, [1.8538, 2.6804])	3.8888 (6.4746, p=0.0000, [2.5779, 5.8662])
Llama 2-13B	1.6950 (5.6197, p=0.0000, [1.4101, 2.0375])	5.6902 (7.6831, p=0.0000, [3.6517, 8.8667])
Llama 2-70B	2.1208 (8.0711, p=0.0000, [1.7669, 2.5456])	4.6884 (7.9747, p=0.0000, [3.2071, 6.8540])
Gemma-7B	1.5294 (4.6095, p=0.0000, [1.2766, 1.8323])	8.4331 (8.7017, p=0.0000, [5.2170, 13.6318])
Gemma-7B-IT	0.9431 (-0.5996, p=0.5488, [0.7787, 1.1422])	8.6569 (7.9881, p=0.0000, [5.0976, 14.7011])

3.6. Supplementary Table 6: Study 1: Ingroup solidarity and outgroup hostility of pre-training datasets using a Random Subsample

Each value represents an odds ratio from a separate logistic regression fitted on a total of 4,000 ingroup and outgroup sentences from a specific corpus predicting whether the sentence is positive (for ingroup solidarity) based on whether the sentence is ingroup (vs. outgroup), and negative (for outgroup hostility) based on whether the sentence is outgroup (vs. ingroup), and control variables. Tests are two-sided, and p-values are not adjusted for multiple comparisons. Sentence sentiment determined using RoBERTa. See Methods for more details. Data are presented as OR (z value, p value, 95% CI).

	Ingroup Solidarity	Outgroup Hostility
C4	1.6687 (7.8802, p=0.0000, [1.4692, 1.8954])	2.1939 (7.0454, p=0.0000, [1.7631, 2.7298])
GPT2	1.9335 (8.5515, p=0.0000, [1.6624, 2.2490])	1.6739 (7.1241, p=0.0000, [1.4527, 1.9288])
OLM	1.3516 (4.5975, p=0.0000, [1.1887, 1.5369])	1.7754 (5.5002, p=0.0000, [1.4470, 2.1784])
The Pile	1.9549 (9.2044, p=0.0000, [1.6948, 2.2548])	1.4457 (4.5676, p=0.0000, [1.2342, 1.6934])

3.7. Supplementary Table 7: Study 1: Ingroup Solidarity and Outgroup Hostility of Pre-training Datasets using the Full Datasets

Each value represents an odds ratio from a separate logistic regression fitted on all ingroup and outgroup sentences from a specific corpus predicting whether the sentence is positive (for ingroup solidarity) based on whether the sentence is ingroup (vs. outgroup), and negative (for outgroup hostility) based on whether the sentence is outgroup (vs. ingroup), and control variables. Tests are two-sided, and p-values are not adjusted for multiple comparisons. Sentence sentiment determined using RoBERTa. A total of 770,302 sentences with 23,461 from C4, 55,520 from GPT2, 677,938 from OLM, and 13,383 from the Pile. See Methods for more details. Data are presented as OR (z value, p value, 95% CI).

	Ingroup Solidarity	Outgroup Hostility
C4	1.5638 (16.4894, p=0.0000, [1.4829, 1.6491])	2.0508 (16.0755, p=0.0000, [1.8788, 2.2384])
GPT2	1.8790 (30.6861, p=0.0000, [1.8048, 1.9562])	1.5414 (21.8977, p=0.0000, [1.4829, 1.6023])
OLM	1.3493 (59.2791, p=0.0000, [1.3360, 1.3627])	1.8454 (75.8071, p=0.0000, [1.8164, 1.8749])
The Pile	1.9924 (17.2927, p=0.0000, [1.8426, 2.1543])	1.5614 (9.7940, p=0.0000, [1.4282, 1.7071])

3.8. Supplementary Table 8: Study 1: Ingroup solidarity and outgroup hostility of a subset base LLMs controlling for sentence topic (Part 1).

Each value represents an odds ratio from a separate logistic regression fitted on a total of 2,000 ingroup and outgroup sentences from a specific LLM predicting whether the sentence is positive (for ingroup solidarity) based on whether the sentence is ingroup (vs. outgroup), and negative (for outgroup hostility) based on whether the sentence is outgroup (vs. ingroup), and control variables. Tests are two-sided, and p-values are not adjusted for multiple comparisons. We consider the results statistically significant if p <.0004, obtained by dividing .05 by the total number of tests with the default prompt (112). Sentence sentiment determined using RoBERTa. See Supplementary Section 4 for more details. Data are presented as OR (z value, p value, 95% CI).

	Ingroup Solidarity	Outgroup Hostility
GPT-2-124M	2.5704 (8.3054, p=0.0000, [2.0571, 3.2119])	2.0487 (5.3960, p=0.0000, [1.5789, 2.6584])
GPT-2-Medium-355M	2.3036 (7.6316, p=0.0000, [1.8592, 2.8542])	1.9541 (5.2462, p=0.0000, [1.5214, 2.5098])
GPT-2-Large-774M	2.0751 (6.6142, p=0.0000, [1.6715, 2.5763])	2.3743 (6.6467, p=0.0000, [1.8399, 3.0639])
GPT-2-XL-1.5B	2.0849 (6.6085, p=0.0000, [1.6767, 2.5926])	1.5727 (3.5976, p=0.0003, [1.2289, 2.0126])
davinci	1.8896 (5.7696, p=0.0000, [1.5223, 2.3457])	1.7212 (4.5102, p=0.0000, [1.3594, 2.1792])
text-davinci-003	2.5836 (8.6667, p=0.0000, [2.0845, 3.2022])	1.8064 (3.6217, p=0.0003, [1.3117, 2.4876])
BLOOM-560M	1.4186 (3.2886, p=0.0010, [1.1517, 1.7472])	1.4416 (2.2256, p=0.0260, [1.0446, 1.9895])
BLOOM-1B1	1.3053 (2.4030, p=0.0163, [1.0503, 1.6220])	1.8093 (3.5449, p=0.0004, [1.3036, 2.5112])
BLOOM-1B7	2.2756 (7.1905, p=0.0000, [1.8187, 2.8473])	2.2273 (4.5646, p=0.0000, [1.5792, 3.1412])
BLOOM-3B	1.5742 (4.0589, p=0.0000, [1.2645, 1.9599])	1.5701 (2.8948, p=0.0038, [1.1568, 2.1309])
BLOOMZ-560M	0.8613 (-1.3213, p=0.1864, [0.6901, 1.0749])	1.1399 (0.7525, p=0.4517, [0.8105, 1.6033])
BLOOMZ-1B1	1.7083 (4.7847, p=0.0000, [1.3718, 2.1272])	1.0944 (0.5188, p=0.6039, [0.7783, 1.5389])
BLOOMZ-1B7	1.5180 (3.7171, p=0.0002, [1.2181, 1.8916])	1.1824 (1.0593, p=0.2895, [0.8672, 1.6122])
BLOOMZ-3B	1.5026 (3.6577, p=0.0003, [1.2081, 1.8690])	1.0967 (0.5702, p=0.5685, [0.7986, 1.5059])
LLaMA-7B	2.0111 (6.0481, p=0.0000, [1.6036, 2.5221])	2.7857 (4.1980, p=0.0000, [1.7267, 4.4944])
LLaMA-13B	2.3873 (7.6113, p=0.0000, [1.9081, 2.9869])	5.2322 (5.9899, p=0.0000, [3.0446, 8.9918])
LLaMA-33B	2.1827 (6.8648, p=0.0000, [1.7466, 2.7276])	3.9186 (5.2406, p=0.0000, [2.3513, 6.5307])
LLaMA-65B	2.0903 (6.5103, p=0.0000, [1.6742, 2.6098])	4.0931 (5.0026, p=0.0000, [2.3564, 7.1097])
Llama 2-7B	2.2017 (6.7375, p=0.0000, [1.7500, 2.7699])	2.3609 (3.5932, p=0.0003, [1.4777, 3.7721])
Llama 2-13B	1.7362 (4.9306, p=0.0000, [1.3943, 2.1620])	5.7883 (6.6976, p=0.0000, [3.4626, 9.6761])
Llama 2-70B	2.1117 (6.7459, p=0.0000, [1.6995, 2.6239])	3.2610 (5.2033, p=0.0000, [2.0892, 5.0901])
OPT-125M	1.7399 (5.1320, p=0.0000, [1.4082, 2.1498])	1.6846 (4.2237, p=0.0000, [1.3225, 2.1458])
OPT-350M	2.1066 (6.5684, p=0.0000, [1.6867, 2.6311])	1.6313 (4.1069, p=0.0000, [1.2915, 2.0604])
OPT-1.3B	1.7906 (5.0326, p=0.0000, [1.4271, 2.2466])	1.3974 (2.7575, p=0.0058, [1.1016, 1.7726])
OPT-2.7B	2.1958 (6.8262, p=0.0000, [1.7519, 2.7521])	1.5143 (3.5742, p=0.0004, [1.2061, 1.9012])
OPT-6.7B	2.6493 (8.4999, p=0.0000, [2.1163, 3.3167])	2.0387 (6.0631, p=0.0000, [1.6194, 2.5666])

3.9. Supplementary Table 9: Study 1: Ingroup solidarity and outgroup hostility of a subset base LLMs controlling for sentence topic (Part 2).

Each value represents an odds ratio from a separate logistic regression fitted on a total of 2,000 ingroup and outgroup sentences from a specific LLM predicting whether the sentence is positive (for ingroup solidarity) based on whether the sentence is ingroup (vs. outgroup), and negative (for outgroup hostility) based on whether the sentence is outgroup (vs. ingroup), and control variables. Tests are two-sided, and p-values are not adjusted for multiple comparisons. We consider the results statistically significant if $p < .0004$, obtained by dividing .05 by the total number of tests with the default prompt (112). Sentence sentiment determined using RoBERTa. See Supplementary Section 4 for more details. Data are presented as OR (z value, p value, 95% CI).

	Ingroup Solidarity	Outgroup Hostility
OPT-13B	1.7950 (5.1764, p=0.0000, [1.4383, 2.2400])	1.4778 (3.3579, p=0.0008, [1.1766, 1.8562])
OPT-30B	1.9815 (5.9337, p=0.0000, [1.5809, 2.4837])	1.6861 (4.4883, p=0.0000, [1.3422, 2.1182])
OPT-66B	1.8514 (5.3023, p=0.0000, [1.4744, 2.3247])	1.5120 (3.5584, p=0.0004, [1.2041, 1.8987])
OPT-IML-1.3B	1.7539 (4.8664, p=0.0000, [1.3987, 2.1992])	1.2294 (1.8894, p=0.0588, [0.9923, 1.5232])
OPT-IML-30B	2.5518 (7.8295, p=0.0000, [2.0184, 3.2263])	1.9467 (5.6393, p=0.0000, [1.5444, 2.4538])
Pythia-70M	2.0811 (5.7401, p=0.0000, [1.6203, 2.6728])	2.9756 (7.5921, p=0.0000, [2.2455, 3.9430])
Pythia-160M	2.4472 (7.9101, p=0.0000, [1.9605, 3.0547])	2.3370 (6.5293, p=0.0000, [1.8113, 3.0152])
Pythia-410M	1.9345 (6.0244, p=0.0000, [1.5608, 2.3977])	1.8501 (4.6510, p=0.0000, [1.4276, 2.3977])
Pythia-1B	2.2134 (6.8085, p=0.0000, [1.7609, 2.7823])	2.0188 (5.1040, p=0.0000, [1.5415, 2.6440])
Pythia-1.4B	1.9490 (6.0771, p=0.0000, [1.5716, 2.4170])	1.7851 (4.2404, p=0.0000, [1.3657, 2.3333])
Pythia-2.8B	2.2830 (7.3213, p=0.0000, [1.8304, 2.8477])	1.8585 (4.6686, p=0.0000, [1.4327, 2.4108])
Pythia-6.9B	2.1809 (6.9430, p=0.0000, [1.7500, 2.7179])	2.0919 (5.0152, p=0.0000, [1.5678, 2.7914])
Pythia-12B	2.1730 (6.6860, p=0.0000, [1.7308, 2.7282])	1.9383 (4.9306, p=0.0000, [1.4899, 2.5215])
Dolly2.0-3B	2.3454 (7.2934, p=0.0000, [1.8652, 2.9492])	2.5318 (6.6861, p=0.0000, [1.9283, 3.3243])
Dolly2.0-7B	1.9199 (5.7579, p=0.0000, [1.5377, 2.3973])	1.6093 (3.2439, p=0.0012, [1.2072, 2.1452])
Dolly2.0-12B	2.1714 (6.8955, p=0.0000, [1.7419, 2.7068])	2.1445 (5.2717, p=0.0000, [1.6149, 2.8478])
Cerebras-GPT-111M	1.3959 (2.8363, p=0.0046, [1.1086, 1.7577])	2.0922 (5.1950, p=0.0000, [1.5836, 2.7641])
Cerebras-GPT-256M	1.4692 (3.2220, p=0.0013, [1.1626, 1.8566])	1.5806 (3.6040, p=0.0003, [1.2322, 2.0274])
Cerebras-GPT-590M	2.6120 (8.6380, p=0.0000, [2.1007, 3.2478])	1.9029 (4.4116, p=0.0000, [1.4298, 2.5325])
Cerebras-GPT-1.3B	2.2583 (7.6208, p=0.0000, [1.8315, 2.7847])	1.8609 (4.5358, p=0.0000, [1.4229, 2.4338])
Cerebras-GPT-2.7B	1.8120 (5.4273, p=0.0000, [1.4620, 2.2460])	2.4665 (6.6644, p=0.0000, [1.8913, 3.2165])
Cerebras-GPT-6.7B	2.5412 (8.2286, p=0.0000, [2.0350, 3.1733])	1.4779 (2.9183, p=0.0035, [1.1369, 1.9213])
Cerebras-GPT-13B	2.0731 (6.4695, p=0.0000, [1.6622, 2.5854])	2.2849 (5.7921, p=0.0000, [1.7276, 3.0221])
J2-Jumbo-Instruct	1.7981 (5.4709, p=0.0000, [1.4572, 2.2186])	1.7931 (3.0996, p=0.0019, [1.2395, 2.5939])
text-bison@001	1.1015 (0.8534, p=0.3935, [0.8822, 1.3753])	0.9248 (-0.4050, p=0.6855, [0.6335, 1.3501])

3.10. Supplementary Table 10: Study 1: Mixed effects logistic regression results showing overall social identity biases for models tested with the default prompt.

Two mixed effects logistic regressions fitted on ingroup and outgroup sentences predicting whether the sentence is positive (for ingroup solidarity) based on whether the sentence is ingroup (vs. outgroup), and negative (for outgroup hostility) based on whether the sentence is outgroup (vs. ingroup), and control variables, with model (such as GPT-2-124M) as random effect. Tests are two-sided, and p-values are not adjusted for multiple comparisons. Sentence sentiment determined using RoBERTa.

For the "Positive" model, the odds ratio of source [with we sentences as the reference group] reflects ingroup solidarity. For the "Negative" model, the odds ratio of source [with they sentences as the reference group] reflects outgroup hostility. See Methods for details; p-values estimated using sjPlot with infinite degrees of freedom.

	Positive				Negative			
Predictors	*Odds Ratios*	*95% CI*	*Z Value*	*p*	*Odds Ratios*	*95% CI*	*Z Value*	*p*
(Intercept)	0.24	0.20 – 0.29	-15.26	**1.3691e-52**	0.13	0.10 – 0.16	-16.57	**1.1139e-61**
source [we]	1.93	1.89 – 1.98	51.76	**0.0000e+00**				
total tokens scaled	1.13	1.11 – 1.15	16.10	**2.6939e-58**	0.96	0.94 – 0.98	-4.06	**4.9630e-05**
TTR	1.98	1.67 – 2.35	7.95	**1.8366e-15**	0.96	0.77 – 1.19	-0.41	6.7976e-01
source [they]					2.15	2.08 – 2.23	44.90	**0.0000e+00**
**Random Effects**								
σ^2	3.29				3.29			
τ_{00}	0.13 $_{model}$				0.27 $_{model}$			
ICC	0.04				0.08			
N	56 $_{model}$				56 $_{model}$			
Observations	112000				112000			
Marginal R^2/ Conditional R^2	0.037 / 0.073				0.041 / 0.114			

3.11. Supplementary Table 11: Study 1: Mixed effects logistic regression results showing the effect of model size on social identity biases.

Two mixed effects logistic regressions fitted on ingroup and outgroup sentences predicting whether the sentence is positive (for ingroup solidarity) based on whether the sentence is ingroup (vs. outgroup), and negative (for outgroup hostility) based on whether the sentence is outgroup (vs. ingroup), and control variables, with model family (such as GPT-2) as random effect. Tests are two-sided, and p-values are not adjusted for multiple comparisons. Sentence sentiment determined using RoBERTa.

For the "Positive" model, the odds ratio of "source [with we sentences as the reference group] × model size scaled" reflects that ingroup solidarity gets larger as the size of the model grows if OR>1. For the "Negative" model, the odds ratio of "source [with they sentences as the reference group] × model size scaled" reflects that outgroup hostility gets larger as the size of the model grows if OR>1. See Methods for details; p-values estimated using sjPlot with infinite degrees of freedom.

	**Positive**				**Negative**			
Predictors	*Odds Ratios*	*95% CI*	*Z Value*	*p*	*Odds Ratios*	*95% CI*	*Z Value*	*p*
(Intercept)	0.25	0.20 – 0.33	-10.75	**5.9184e-27**	0.11	0.08 – 0.16	-11.82	**3.2463e-32**
source [we]	1.93	1.88 – 1.98	48.58	**0.0000e+00**				
total tokens scaled	1.14	1.12 – 1.16	16.52	**2.6235e-61**	0.96	0.94 – 0.98	-4.04	**5.2607e-05**
TTR	2.10	1.76 – 2.52	8.19	**2.5934e-16**	0.97	0.78 – 1.21	-0.26	7.9365e-01
model size scaled	0.98	0.96 – 0.99	-2.75	**6.0126e-03**	0.97	0.95 – 0.99	-2.39	**1.6729e-02**
source [we] × model size scaled	1.02	1.00 – 1.04	2.19	**2.8473e-02**				
source [they]					2.15	2.08 – 2.23	42.83	**0.0000e+00**
source [they] × model size scaled					1.08	1.05 – 1.11	5.13	**2.9652e-07**
**Random Effects**								
σ^2	3.29				3.29			
τ_{00}	0.12 $_{model.family}$				0.31 $_{model.family}$			
ICC	0.03				0.09			
N	13 $_{model.family}$				13 $_{model.family}$			
Observations	104000				104000			
Marginal R^2/ Conditional R^2	0.038 / 0.071				0.043 / 0.125			

3.12. Supplementary Table 12: Study 1: Ingroup solidarity and outgroup hostility of LLMs tested with the instruction prompt.

Each value represents an odds ratio from a separate logistic regression fitted on a total of 2,000 ingroup and outgroup sentences from a specific LLM predicting whether the sentence is positive (for ingroup solidarity) based on whether the sentence is ingroup (vs. outgroup), and negative (for outgroup hostility) based on whether the sentence is outgroup (vs. ingroup), and control variables. Tests are two-sided, and p-values are not adjusted for multiple comparisons. We consider the results statistically significant if p <.0004, obtained by dividing .05 by the total number of tests with the default prompt (112). Sentence sentiment determined using RoBERTa. See Methods for more details. Data are presented as OR (z value, p value, 95% CI).

	Ingroup Solidarity	Outgroup Hostility
GPT-4	1.0362 (0.3717, p=0.7101, [0.8592, 1.2496])	1.0027 (0.0153, p=0.9878, [0.7087, 1.4186])
text-davinci-003	1.2942 (2.7254, p=0.0064, [1.0751, 1.5580])	1.5875 (2.5890, p=0.0096, [1.1188, 2.2526])
Llama 2-7B-chat	1.3755 (3.4122, p=0.0006, [1.1453, 1.6519])	0.7880 (-1.6983, p=0.0895, [0.5985, 1.0374])
Llama 2-13B-chat	1.3900 (3.5305, p=0.0004, [1.1578, 1.6689])	0.9408 (-0.4145, p=0.6785, [0.7052, 1.2553])
Llama 2-70B-chat	1.1152 (1.1783, p=0.2387, [0.9302, 1.3369])	0.7193 (-2.3257, p=0.0200, [0.5449, 0.9495])
Dolly2.0-3B	1.1917 (1.8369, p=0.0662, [0.9883, 1.4370])	1.8937 (4.0677, p=0.0000, [1.3922, 2.5758])
Dolly2.0-7B	0.9769 (-0.2570, p=0.7972, [0.8174, 1.1675])	1.4258 (2.4222, p=0.0154, [1.0700, 1.9000])
Dolly2.0-12B	0.9924 (-0.0845, p=0.9327, [0.8306, 1.1856])	1.9575 (4.1962, p=0.0000, [1.4304, 2.6789])
Flan-T5-XL-3B	1.9523 (6.9846, p=0.0000, [1.6182, 2.3555])	1.4922 (2.4172, p=0.0156, [1.0786, 2.0642])
Flan-T5-XXL-11B	1.6313 (5.1695, p=0.0000, [1.3551, 1.9640])	1.1362 (0.7863, p=0.4317, [0.8265, 1.5619])
Flan-UL2-20B	2.2327 (8.0907, p=0.0000, [1.8379, 2.7123])	1.4649 (2.5511, p=0.0107, [1.0925, 1.9643])
text-bison@001	1.5454 (4.6993, p=0.0000, [1.2888, 1.8530])	1.0290 (0.1723, p=0.8632, [0.7430, 1.4253])
chat-bison@001	1.3802 (3.5160, p=0.0004, [1.1533, 1.6517])	1.4628 (2.3073, p=0.0210, [1.0589, 2.0206])
OLMo-7B-Instruct	1.7664 (5.7431, p=0.0000, [1.4547, 2.1449])	1.3843 (1.6765, p=0.0936, [0.9465, 2.0246])
OLMo-7B-SFT	1.7235 (5.8196, p=0.0000, [1.4348, 2.0702])	0.9702 (-0.1843, p=0.8537, [0.7032, 1.3385])
Tulu-2-7B	1.2682 (2.5713, p=0.0101, [1.0581, 1.5200])	0.9927 (-0.0471, p=0.9625, [0.7315, 1.3472])
Tulu-2-13B	1.6285 (5.3480, p=0.0000, [1.3620, 1.9473])	0.9575 (-0.2831, p=0.7771, [0.7089, 1.2933])
Tulu-2-70B	1.3909 (3.6202, p=0.0003, [1.1634, 1.6630])	0.9299 (-0.4750, p=0.6348, [0.6889, 1.2552])
Tulu-2-DPO-7B	1.3874 (3.4456, p=0.0006, [1.1516, 1.6714])	1.5333 (2.4994, p=0.0124, [1.0966, 2.1439])
Tulu-2-DPO-13B	1.0410 (0.4028, p=0.6871, [0.8560, 1.2661])	0.6661 (-2.1793, p=0.0293, [0.4623, 0.9599])
Tulu-2-DPO-70B	0.9217 (-0.8736, p=0.3823, [0.7676, 1.1067])	0.7881 (-1.3652, p=0.1722, [0.5599, 1.1093])
J2-Jumbo-Instruct	1.4500 (3.9999, p=0.0001, [1.2086, 1.7395])	1.1414 (0.9306, p=0.3520, [0.8639, 1.5079])
Alpaca-7B	1.2832 (2.7297, p=0.0063, [1.0729, 1.5348])	1.1403 (0.7867, p=0.4315, [0.8222, 1.5815])
Zephyr-7B-beta	1.1414 (1.4641, p=0.1432, [0.9562, 1.3625])	0.8928 (-0.6864, p=0.4925, [0.6460, 1.2341])
Starling-7B	1.1288 (1.3080, p=0.1909, [0.9414, 1.3536])	0.7337 (-1.8727, p=0.0611, [0.5306, 1.0145])
OpenChat3.5-7B	0.9694 (-0.3391, p=0.7345, [0.8099, 1.1603])	0.6844 (-2.2118, p=0.0270, [0.4891, 0.9577])
Gemma-7B-IT	1.3204 (3.0010, p=0.0027, [1.1012, 1.5832])	1.2680 (1.3732, p=0.1697, [0.9035, 1.7794])
Mixtral-8x7B-Instruct	1.1369 (1.4080, p=0.1591, [0.9509, 1.3593])	0.9904 (-0.0578, p=0.9539, [0.7143, 1.3732])

3.13. Supplementary Table 13: Study 1: Mixed effects logistic regression results showing the effects of instructions fine-tuning on social identity biases tested with the default prompt.

Two mixed effects logistic regressions fitted on ingroup and outgroup sentences predicting whether the sentence is positive (for ingroup solidarity) based on whether the sentence is ingroup (vs. outgroup), and negative (for outgroup hostility) based on whether the sentence is outgroup (vs. ingroup), and control variables, with base model (such as BLOOM-1B1 for BLOOM-1B1 and BLOOMZ-1B1) as random effect. Tests are two-sided, and p-values are not adjusted for multiple comparisons. Sentence sentiment determined using RoBERTa. See Supplementary Table 15 for the base-instruction model correspondence.

For the "Positive" model, the odds ratio of "source [with we sentences as the reference group] × instruction fine tuned" reflects that ingroup solidarity is higher for instruction fine-tuned models than base models if OR>1. For the "Negative" model, the odds ratio of "source [with they sentences as the reference group] × instruction fine tuned" reflects that outgroup hostility is higher for instruction fine-tuned models than base models if OR>1. See Methods for details; p-values estimated using sjPlot with infinite degrees of freedom.

	**Positive**				**Negative**			
Predictors	*Odds Ratios*	*95% CI*	*Z Value*	*p*	*Odds Ratios*	*95% CI*	*Z Value*	*p*
(Intercept)	0.22	0.17 – 0.30	-9.83	**8.7723e-23**	0.11	0.07 – 0.18	-8.90	**5.4642e-19**
source [we]	1.72	1.51 – 1.95	8.39	**4.8477e-17**				
total tokens scaled	1.18	1.15 – 1.21	12.71	**4.9712e-37**	0.94	0.91 – 0.97	-3.69	**2.2594e-04**
TTR	3.07	2.30 – 4.10	7.58	**3.3914e-14**	0.99	0.68 – 1.44	-0.05	9.6312e-01
instruction fine tuned	0.81	0.76 – 0.86	-6.66	**2.7331e-11**	1.12	1.03 – 1.22	2.53	**1.1426e-02**
source [we] × instruction fine tuned	1.02	0.93 – 1.10	0.36	7.2025e-01				
source [they]					2.92	2.45 – 3.46	12.14	**6.6816e-34**
source [they] × instruction fine tuned					0.76	0.68 – 0.85	-4.77	**1.8044e-06**
**Random Effects**								
σ^2	3.29				3.29			
τ_{00}	0.03 $_{model.instruct}$				0.26 $_{model.instruct}$			
ICC	0.01				0.07			
N	10 $_{model.instruct}$				10 $_{model.instruct}$			
Observations	40000				40000			
Marginal R^2/ Conditional R^2	0.035 / 0.043				0.036 / 0.105			

3.14. Supplementary Table 14: Study 1: Mixed effects logistic regression results showing the effects of preference fine-tuning on social identity biases tested with the instruction prompt.

Two mixed effects logistic regressions fitted on ingroup and outgroup sentences predicting whether the sentence is positive (for ingroup solidarity) based on whether the sentence is ingroup (vs. outgroup), and negative (for outgroup hostility) based on whether the sentence is outgroup (vs. ingroup), and control variables, with base model (such as BLOOM-1B1 for BLOOM-1B1 and BLOOMZ-1B1) as random effect. Tests are two-sided, and p-values are not adjusted for multiple comparisons. Sentence sentiment determined using RoBERTa. Base models were tested with the default prompt while the preference fine-tuned models were tested with the instruction prompt. See Supplementary Table 15 for the base-preference model correspondence.

For the "Positive" model, the odds ratio of "source [with we sentences as the reference group] × preference fine tuned" reflects that ingroup is higher for preference fine-tuned models than base models if OR>1. For the "Negative" model, the odds ratio of "source [with they sentences as the reference group] × preference fine tuned" reflects that outgroup is higher for preference fine-tuned models than base models if OR>1. See Methods for details; p-values estimated using sjPlot with infinite degrees of freedom.

	**Positive**				**Negative**			
Predictors	*Odds Ratios*	*95% CI*	*Z Value*	*p*	*Odds Ratios*	*95% CI*	*Z Value*	*p*
(Intercept)	0.12	0.08 – 0.17	-11.05	**2.1369e-28**	0.05	0.03 – 0.10	-9.19	**3.7844e-20**
source [we]	1.93	1.79 – 2.08	17.47	**2.6172e-68**				
total tokens scaled	1.23	1.19 – 1.27	12.98	**1.4911e-38**	0.94	0.90 – 1.00	-2.13	**3.3422e-02**
TTR	7.70	5.18 – 11.44	10.10	**5.7878e-24**	0.83	0.43 – 1.58	-0.57	5.6708e-01
preference fine tuned	1.72	1.61 – 1.84	15.64	**4.1914e-55**	2.37	2.05 – 2.74	11.61	**3.5580e-31**
source [we] × preference fine tuned	0.61	0.55 – 0.67	-10.25	**1.2090e-24**				
source [they]					3.79	3.28 – 4.38	17.99	**2.4293e-72**
source [they] × preference fine tuned					0.22	0.18 – 0.26	-16.59	**8.7780e-62**
**Random Effects**								
σ^2	3.29				3.29			
τ_{00}	0.02 $_{model.preference}$				0.00 $_{model.preference}$			
ICC	0.00				0.00			
N	6 $_{model.preference}$				6 $_{model.preference}$			
Observations	30000				30000			
Marginal R^2/ Conditional R^2	0.034 / 0.039				0.054 / 0.055			

3.15. Supplementary Table 15: Correspondence between base models, instruction-tuned models, and preference-tuned models.

Models prompted with the instruction prompt only are marked with (i) and were compared with their base versions prompted with the default prompt. API-based models are excluded due to unclear correspondences. For instruction-tuned models, we only compared those prompted with the default prompt for maximal correspondence. See Methods for more details.

Base Model Family	Base Model	Instruction-Tuned Model	Preference-Tuned Model
BLOOM	BLOOM-560M	BLOOMZ-560M	-
	BLOOM-1B1	BLOOMZ-1B1	-
	BLOOM-1B7	BLOOMZ-1B7	-
	BLOOM-3B	BLOOMZ-3B	-
OPT	OPT-1.3B	OPT-IML-1.3B	-
	OPT-30B	OPT-IML-30B	-
Pythia	Pythia-2.8B	Dolly 2.0-3B	-
	Pythia-6.9B	Dolly 2.0-7B	-
	Pythia-12B	Dolly 2.0-12B	-
Gemma	Gemma-7B	Gemma-7B-IT	-
LLaMA 2	LLaMA 2-7B	-	LLaMA 2-7B-Chat (i)
	LLaMA 2-13B	-	LLaMA 2-13B-Chat (i)
	LLaMA 2-70B	-	LLaMA 2-70B-Chat (i)
	LLaMA 2-7B	Tulu 2-7B (i)	Tulu 2-DPO-7B (i)
	LLaMA 2-13B	Tulu 2-13B (i)	Tulu 2-DPO-13B (i)
	LLaMA 2-70B	Tulu 2-70B (i)	Tulu 2-DPO-70B (i)
OLMo	OLMo-7B	OLMo-7B-SFT (i)	OLMo-7B-Instruct (i)
LLaMA	LLaMA-7B	Alpaca-7B (i)	-
Mistral	Mistral-7B	Zephyr-7B-Beta (i)	-
	Mistral-7B	-	Starling-7B (i)
	Mixtral-8x7B	Mixtral-8x7B-Instruct (i)	-
	Mistral-7B	-	OpenChat3.5-7B (i)

3.16. Supplementary Table 16: Sentiment analysis results across three classifiers with and without swapping “We” for “They” and vice versa.

See Supplementary Section 5 for details.

**RoBERTa**	**Negative**	**Neutral**	**Positive**
We	1613	3775	4612
We → They	1729	4162	4109
They	3623	3707	2670
They → We	3289	3758	2953
**DynaSent**	**Negative**	**Neutral**	**Positive**
We	1628	1801	6571
We → They	1644	2076	6280
They	3237	2692	4071
They → We	2905	2562	4533
**GPT-4o-mini**	**Negative**	**Neutral**	**Positive**
We	1125	4840	4035
We → They	1082	4957	3961
They	2811	4870	2319
They → We	2738	5089	2173

3.17. Supplementary Table 17: Study 2: Mixed effects logistic regression results showing overall social identity biases for the partisan fine-tuned models.

Two mixed effects logistic regressions fitted on ingroup and outgroup sentences predicting whether the sentence is positive (for ingroup solidarity) based on whether the sentence is ingroup (vs. outgroup), and negative (for outgroup hostility) based on whether the sentence is outgroup (vs. ingroup), and control variables, with model (such as GPT-2-124M) and fine-tuning party (such as Republican or Democrat) as random effects. Tests are two-sided, and p-values are not adjusted for multiple comparisons. Sentence sentiment determined using RoBERTa.

For the “Positive” model, the odds ratio of source [with we sentences as the reference group] reflects ingroup solidarity. For the “Negative” model, the odds ratio of source [with they sentences as the reference group] reflects outgroup hostility. See Methods for details; p-values estimated using sjPlot with infinite degrees of freedom.

	Positive				Negative			
Predictors	*Odds Ratios*	*95% CI*	*Z Value*	*p*	*Odds Ratios*	*95% CI*	*Z Value*	*p*
(Intercept)	0.10	0.08 – 0.13	-19.39	**8.7672e-84**	0.18	0.14 – 0.22	-15.93	**3.7246e-57**
source [we]	4.61	4.42 – 4.82	69.29	**0.0000e+00**				
total tokens scaled	1.05	1.02 – 1.08	3.44	**5.7817e-04**	1.17	1.14 – 1.20	11.23	**2.7525e-29**
TTR	1.91	1.50 – 2.43	5.24	**1.6310e-07**	1.51	1.20 – 1.90	3.55	**3.8956e-04**
source [they]					6.50	6.24 – 6.78	88.98	**0.0000e+00**
**Random Effects**								
σ^2	3.29				3.29			
τ_{00}	0.01 $_{model}$				0.00 $_{model}$			
	0.00 $_{fine.tuned.party}$				0.00 $_{fine.tuned.party}$			
ICC	0.00							
N	12 $_{model}$				12 $_{model}$			
	2 $_{fine.tuned.party}$				2 $_{fine.tuned.party}$			
Observations	48000				48000			
Marginal R^2/ Conditional R^2	0.152 / 0.155				0.210 / NA			

3.18. Supplementary Table 18: Study 2: Mixed effects logistic regression results showing overall social identity biases for base models before partisan fine-tuning.

Two mixed effects logistic regressions fitted on ingroup and outgroup sentences predicting whether the sentence is positive (for ingroup solidarity) based on whether the sentence is ingroup (vs. outgroup), and negative (for outgroup hostility) based on whether the sentence is outgroup (vs. ingroup), and control variables, with model (such as GPT-2-124M) as random effect. Tests are two-sided, and p-values are not adjusted for multiple comparisons. Sentence sentiment determined using RoBERTa.

For the "Positive" model, the odds ratio of source [with we sentences as the reference group] reflects ingroup solidarity. For the "Negative" model, the odds ratio of source [with they sentences as the reference group] reflects outgroup hostility. See Methods for details; p-values estimated using sjPlot with infinite degrees of freedom.

	**Positive**				**Negative**			
Predictors	*Odds Ratios*	*95% CI*	*Z Value*	*p*	*Odds Ratios*	*95% CI*	*Z Value*	*p*
(Intercept)	0.23	0.16 – 0.33	-7.76	**8.2037e-15**	0.11	0.07 – 0.18	-8.51	**1.6947e-17**
source [we]	1.86	1.76 – 1.96	22.70	**4.9255e-114**				
total tokens scaled	1.11	1.07 – 1.14	6.04	**1.5150e-09**	0.91	0.87 – 0.95	-4.23	**2.2989e-05**
TTR	2.13	1.46 – 3.10	3.95	**7.9361e-05**	1.13	0.69 – 1.88	0.49	6.2496e-01
source [they]					1.83	1.69 – 1.97	15.86	**1.1232e-56**
**Random Effects**								
σ^2	3.29				3.29			
τ_{00}	0.06 $_{model}$				0.13 $_{model}$			
ICC	0.02				0.04			
N	12 $_{model}$				12 $_{model}$			
Observations	24000				24000			
Marginal R^2/ Conditional R^2	0.032 / 0.048				0.031 / 0.067			

3.19. Supplementary Table 19: Study 2: Mixed effects logistic regression results showing the effect of partisan fine-tuning.

Two mixed effects logistic regressions fitted on ingroup and outgroup sentences predicting whether the sentence is positive (for ingroup solidarity) based on whether the sentence is ingroup (vs. outgroup), and negative (for outgroup hostility) based on whether the sentence is outgroup (vs. ingroup), and control variables, with model (such as GPT-2-124M) and fine-tuning party (Republican, Democrat, or None) as random effects. Tests are two-sided, and p-values are not adjusted for multiple comparisons. Sentence sentiment determined using RoBERTa.

For the "Positive" model, the odds ratio of "source [with we sentences as the reference group] × fine tuned TRUE" reflects that ingroup solidarity is higher for fine-tuned models if OR>1. For the "Negative" model, the odds ratio of source [with they sentences as the reference group] reflects that outgroup hostility is higher for fine-tuned models if OR>1. See Methods for details; p-values estimated using sjPlot with infinite degrees of freedom.

	**Positive**				**Negative**			
Predictors	*Odds Ratios*	*95% CI*	*Z Value*	*p*	*Odds Ratios*	*95% CI*	*Z Value*	*p*
(Intercept)	0.25	0.20 – 0.30	-13.84	**1.5278e-43**	0.09	0.07 – 0.11	-23.12	**3.0215e-118**
source [we]	1.86	1.76 – 1.96	22.91	**3.4530e-116**				
total tokens scaled	1.08	1.05 – 1.10	6.91	**4.7935e-12**	1.10	1.08 – 1.13	8.30	**1.0214e-16**
fine tuned TRUE	0.41	0.39 – 0.43	-33.86	**2.2751e-251**	2.13	2.00 – 2.28	22.60	**4.7085e-113**
TTR	1.98	1.61 – 2.42	6.60	**4.0115e-11**	1.41	1.15 – 1.74	3.26	**1.1005e-03**
source [we] × fine tuned TRUE	2.48	2.32 – 2.65	26.13	**1.7867e-150**				
source [they]					1.90	1.76 – 2.04	17.10	**1.5919e-65**
source [they] × fine tuned TRUE					3.40	3.12 – 3.70	28.61	**5.7381e-180**
**Random Effects**								
σ^2	3.29				3.29			
τ_{00}	0.01 $_{model}$				0.01 $_{model}$			
	0.00 $_{fine.tuned}$				0.00 $_{fine.tuned}$			
N	3 $_{fine.tuned}$				3 $_{fine.tuned}$			
	12 $_{model}$				12 $_{model}$			
Observations	72000				72000			
Marginal R^2/ Conditional R^2	0.127 / NA				0.235 / NA			

3.20. Supplementary Table 20: Study 1: Mixed effects logistic regression results showing the overall ingroup solidarity and outgroup hostility biases of sentences from pre-training corpora.

Two mixed effects logistic regressions fitted on ingroup and outgroup sentences predicting whether the sentence is positive (for ingroup solidarity) based on whether the sentence is ingroup (vs. outgroup), and negative (for outgroup hostility) based on whether the sentence is outgroup (vs. ingroup), and control variables, with pretraining corpus as random effect. Tests are two-sided, and p-values are not adjusted for multiple comparisons. Sentence sentiment determined using RoBERTa.

For the "Positive" model, the odds ratio of source [with we sentences as the reference group] reflects ingroup solidarity. For the "Negative" model, the odds ratio of source [with they sentences as the reference group] reflects outgroup hostility. See Methods for details; p-values estimated using sjPlot with infinite degrees of freedom.

	**Positive**				**Negative**			
Predictors	*Odds Ratios*	*95% CI*	*Z Value*	*p*	*Odds Ratios*	*95% CI*	*Z Value*	*p*
(Intercept)	0.14	0.08 – 0.26	-6.23	4.8076e-10	0.26	0.12 – 0.56	-3.47	5.2008e-04
source [we]	1.68	1.57 – 1.80	15.03	4.9448e-51				
total tokens scaled	1.10	1.05 – 1.15	4.43	9.5406e-06	0.94	0.90 – 1.00	-2.10	3.5919e-02
TTR	2.75	1.63 – 4.62	3.80	1.4266e-04	0.52	0.28 – 0.98	-2.02	4.3126e-02
source [they]					1.70	1.56 – 1.85	12.08	1.2922e-33
**Random Effects**								
σ^2	3.29				3.29			
τ_{00}	0.14 $_{corpus}$				0.24 $_{corpus}$			
ICC	0.04				0.07			
N	4 $_{corpus}$				4 $_{corpus}$			
Observations	16000				16000			
Marginal R^2 / Conditional R^2	0.022 / 0.063				0.021 / 0.088			

3.21. Supplementary Table 21: Study 1: Logistic regression results showing the comparison of ingroup and outgroup bias of sentences from pre-training corpora and sentences from LLMs combined.

Two logistic regressions fitted on ingroup and outgroup sentences predicting whether the sentence is positive (for ingroup solidarity) based on whether the sentence is ingroup (vs. outgroup), and negative (for outgroup hostility) based on whether the sentence is outgroup (vs. ingroup), and control variables. Tests are two-sided, and p-values are not adjusted for multiple comparisons. Sentence sentiment determined using RoBERTa.

For the “Positive” model, the odds ratio of source [with we sentences as the reference group] × humanTRUE reflects whether the human data has stronger ingroup solidarity if OR>1. For the “Negative” model, the odds ratio of source [with they sentences as the reference group] × humanTRUE reflects whether the human data has stronger outgroup hostility if OR>1. See Methods for details; p-values estimated using sjPlot with infinite degrees of freedom.

	**Positive**				**Negative**			
Predictors	*Odds Ratios*	*95% CI*	*Z Value*	*p*	*Odds Ratios*	*95% CI*	*Z Value*	*p*
(Intercept)	0.20	0.17 – 0.23	-21.80	2.4416e-105	0.14	0.12 – 0.17	-20.45	5.9359e-93
source [we]	1.89	1.84 – 1.94	50.88	0.0000e+00				
humanTRUE	0.81	0.76 – 0.85	-8.10	5.6110e-16	1.15	1.08 – 1.24	4.03	5.4877e-05
total tokens scaled	1.14	1.13 – 1.16	18.95	4.5346e-80	0.95	0.93 – 0.97	-5.48	4.2278e-08
TTR	2.56	2.19 – 3.00	11.66	2.0928e-31	0.94	0.77 – 1.15	-0.58	5.6143e-01
source [we] × humanTRUE	0.87	0.81 – 0.94	-3.73	1.9324e-04				
source [they]					2.09	2.03 – 2.16	44.06	0.0000e+00
source [they] × humanTRUE					0.80	0.73 – 0.87	-4.94	7.6147e-07
Observations	128000				128000			
R^2 Tjur	0.030				0.018			

3.22. Supplementary Table 22: Study 1: Raw counts of positive and negative valanced ingroup and outgroup sentences for models tested with the default prompt.

Sentiment determined using RoBERTa.

Model	Ingroup Positive	Ingroup Negative	Outgroup Positive	Outgroup Negative
BLOOM-1B1	559	84	434	170
BLOOM-1B7	582	73	340	198
BLOOM-3B	518	98	363	194
BLOOM-560M	504	87	389	133
BLOOMZ-1B1	486	87	328	122
BLOOMZ-1B7	344	109	234	123
BLOOMZ-3B	423	111	292	129
BLOOMZ-560M	351	91	354	102
Cerebras-GPT-1.3B	495	126	300	219
Cerebras-GPT-111M	318	105	264	227
Cerebras-GPT-13B	485	119	317	246
Cerebras-GPT-2.7B	445	129	297	264
Cerebras-GPT-256M	277	157	198	219
Cerebras-GPT-590M	503	102	276	191
Cerebras-GPT-6.7B	456	138	253	221
Dolly2.0-12B	485	127	310	225
Dolly2.0-3B	465	126	272	286
Dolly2.0-7B	453	124	317	196
GPT-2-124M	482	139	271	275
GPT-2-Large-774M	444	135	271	277
GPT-2-Medium-355M	459	159	269	272
GPT-2-XL-1.5B	442	156	261	254
Gemma-7B	596	20	477	147
Gemma-7B-IT	377	16	350	127
J2-Jumbo-Instruct	511	71	341	130
LLaMA-13B	656	20	437	141
LLaMA-33B	650	22	471	126
LLaMA-65B	622	21	426	121
LLaMA-7B	627	38	456	114
Llama 2-13B	636	25	490	133
Llama 2-70B	644	36	446	146
Llama 2-7B	658	31	456	118
Mistral-7B	564	57	394	156
Mixtral-8x7B	577	55	382	174
OLMo-7B	588	52	440	149
OPT-1.3B	423	204	278	306
OPT-125M	448	168	294	317
OPT-13B	418	215	256	361
OPT-2.7B	433	219	236	360
OPT-30B	401	223	245	359
OPT-350M	422	204	252	340
OPT-6.7B	464	192	240	367
OPT-66B	382	219	246	358
OPT-IML-1.3B	351	268	238	360
OPT-IML-30B	421	197	215	383
Pythia-1.4B	479	132	311	227
Pythia-12B	429	144	272	269
Pythia-160M	387	127	208	261
Pythia-1B	482	142	279	245
Pythia-2.8B	461	154	284	254
Pythia-410M	450	143	297	233
Pythia-6.9B	468	114	304	211
Pythia-70M	288	104	166	248
davinci	357	168	219	261
text-bison@001	493	94	499	98
text-davinci-003	517	89	285	164

3.23. Supplementary Table 23: Study 1: Descriptive statistics of various metrics for sentences generated with the default prompt.

Statistic	N	Mean	St. Dev.	Min	Max
VADER Sentiment	112,000	0.207	0.398	-0.990	0.995
TextBlob Sentiment	112,000	0.157	0.293	-1.000	1.000
AFINN Sentiment	112,000	1.263	2.782	-35	36
DynaSent Prob.	112,000	0.772	0.174	0.335	0.998
DynaSent Prob. Swapped	112,000	0.769	0.175	0.334	0.998
RoBERTa Prob.	112,000	0.776	0.150	0.338	0.993
RoBERTa Prob. Swapped	112,000	0.772	0.150	0.341	0.993
VADER Positive	112,000	0.488	0.500	0	1
VADER Negative	112,000	0.142	0.349	0	1
AFINN Positive	112,000	0.470	0.499	0	1
AFINN Negative	112,000	0.137	0.344	0	1
TextBlob Positive	112,000	0.540	0.498	0	1
TextBlob Negative	112,000	0.149	0.356	0	1
RoBERTa Positive	112,000	0.397	0.489	0	1
RoBERTa Negative	112,000	0.169	0.374	0	1
DynaSent Positive	112,000	0.552	0.497	0	1
DynaSent Negative	112,000	0.169	0.375	0	1
RoBERTa Swapped Positive	112,000	0.388	0.487	0	1
RoBERTa Swapped Negative	112,000	0.159	0.365	0	1
DynaSent Swapped Positive	112,000	0.557	0.497	0	1
DynaSent Swapped Negative	112,000	0.160	0.366	0	1
RoBERTa Pooled Positive	112,000	0.393	0.488	0	1
RoBERTa Pooled Negative	112,000	0.164	0.370	0	1
DynaSent Pooled Positive	112,000	0.560	0.496	0	1
DynaSent Pooled Negative	112,000	0.169	0.375	0	1
GPT-4o-mini Positive	112,000	0.336	0.472	0	1
GPT-4o-mini Negative	112,000	0.110	0.312	0	1
LIWC Positive	112,000	0.435	0.496	0	1
LIWC Negative	112,000	0.095	0.293	0	1
LIWC Positive Word Count	112,000	0.727	0.966	0	18
LIWC Negative Word Count	112,000	0.181	0.498	0	12
LIWC Sentiment	112,000	0.547	1.112	-11	18
TTR	112,000	0.916	0.093	0.100	1.000
CTTR	112,000	2.545	0.540	0.394	4.583
Total Tokens	112,000	16.952	8.858	1	74
Ingroup RoBERTa Positive	112,000	0.238	0.426	0	1
Ingroup RoBERTa Negative	112,000	0.059	0.235	0	1
Outgroup RoBERTa Positive	112,000	0.159	0.366	0	1
Outgroup RoBERTa Negative	112,000	0.110	0.313	0	1

3.24. Supplementary Table 24: Study 3: Mixed effects logistic regressions results showing social identity biases of LLM-generated sentences in real-world human-LLM conversations.

Two mixed effects logistic regressions fitted on ingroup and outgroup sentences predicting whether the sentence is positive (for ingroup solidarity) based on whether the sentence is ingroup (vs. outgroup), and negative (for outgroup hostility) based on whether the sentence is outgroup (vs. ingroup), and control variables, with dataset (WildChat and LMSYS) as random effect. Tests are two-sided, and p-values are not adjusted for multiple comparisons. Sentence sentiment determined using RoBERTa.

For the "Positive" model, the odds ratio of source [with we sentences as the reference group] reflects ingroup solidarity. For the "Negative" model, the odds ratio of source [with they sentences as the reference group] reflects outgroup hostility. See Methods for details; p-values estimated using sjPlot with infinite degrees of freedom.

	**Positive**				**Negative**			
Predictors	*Odds Ratios*	*95% CI*	*Z Value*	*p*	*Odds Ratios*	*95% CI*	*Z Value*	*p*
(Intercept)	0.21	0.13 – 0.33	-6.69	**2.2920e-11**	0.18	0.10 – 0.34	-5.30	**1.1616e-07**
source [we]	1.80	1.68 – 1.92	17.19	**3.1996e-66**				
total tokens scaled	1.06	1.02 – 1.10	2.96	**3.0324e-03**	0.79	0.74 – 0.84	-7.71	**1.2236e-14**
TTR	3.03	1.91 – 4.80	4.70	**2.5881e-06**	0.53	0.27 – 1.02	-1.91	5.6358e-02
source [they]					1.57	1.40 – 1.75	7.89	**3.1068e-15**
**Random Effects**								
σ^2	3.29				3.29			
τ_{00}	0.01 $_{dataset}$				0.01 $_{dataset}$			
ICC	0.00				0.00			
N	2 $_{dataset}$				2 $_{dataset}$			
Observations	20753				20753			
Marginal R^2/ Conditional R^2	0.019 / 0.022				0.021 / 0.023			

3.25. Supplementary Table 25: Study 3: Mixed effects logistic regressions results showing social identity biases of user-written sentences in real-world human-LLM conversations.

Two mixed effects logistic regressions fitted on ingroup and outgroup sentences predicting whether the sentence is positive (for ingroup solidarity) based on whether the sentence is ingroup (vs. outgroup), and negative (for outgroup hostility) based on whether the sentence is outgroup (vs. ingroup), and control variables, with dataset (WildChat and LMSYS) as random effect. Tests are two-sided, and p-values are not adjusted for multiple comparisons. Sentence sentiment determined using RoBERTa.

For the "Positive" model, the odds ratio of source [with we sentences as the reference group] reflects ingroup solidarity. For the "Negative" model, the odds ratio of source [with they sentences as the reference group] reflects outgroup hostility. See Methods for details; p-values estimated using sjPlot with infinite degrees of freedom.

	Positive				Negative			
Predictors	*Odds Ratios*	*95% CI*	*Z Value*	*p*	*Odds Ratios*	*95% CI*	*Z Value*	*p*
(Intercept)	0.13	0.05 – 0.35	-4.00	**6.3905e-05**	0.06	0.02 – 0.21	-4.36	**1.3294e-05**
source [we]	1.86	1.64 – 2.13	9.31	**1.2251e-20**				
total tokens scaled	1.12	1.06 – 1.19	3.93	**8.5781e-05**	1.02	0.95 – 1.10	0.54	5.8645e-01
TTR	2.32	0.81 – 6.69	1.56	1.1828e-01	1.72	0.45 – 6.57	0.80	4.2621e-01
source [they]					2.58	2.16 – 3.08	10.44	**1.5524e-25**
**Random Effects**								
σ^2	3.29				3.29			
τ_{00}	0.01 $_{dataset}$				0.00 $_{dataset}$			
ICC	0.00							
N	2 $_{dataset}$				2 $_{dataset}$			
Observations	4641				4641			
Marginal R^2/ Conditional R^2	0.035 / 0.040				0.063 / NA			

3.26. Supplementary Table 26: Study 3: Ingroup solidarity and outgroup hostility of both user-written and LLM-generated sentences in real-world human-LLM conversations.

Each value represents an odds ratio from a separate logistic regression fitted on ingroup and outgroup sentences from a specific corpus predicting whether the sentence is positive (for ingroup solidarity) based on whether the sentence is ingroup (vs. outgroup), and negative (for outgroup hostility) based on whether the sentence is outgroup (vs. ingroup), and control variables. Tests are two-sided, and p-values are not adjusted for multiple comparisons. Sentence sentiment determined using RoBERTa. A total of 25,395 sentences with 10,507 from WildChat Model, 2,453 from WildChat Users, 10,247 from LMSYS Models, and 2,188 from LMSYS Users. See Methods for more details. Data are presented as OR (z value, p value, 95% CI).

	Ingroup Solidarity	Outgroup Hostility
WildChat Model	1.7083 (11.6620, p=0.0000, [1.5612, 1.8691])	1.3966 (4.3561, p=0.0000, [1.2017, 1.6230])
WildChat Users	2.1695 (8.6108, p=0.0000, [1.8188, 2.5877])	2.1962 (6.5003, p=0.0000, [1.7324, 2.7842])
LMSYS Model	1.9158 (12.7089, p=0.0000, [1.7330, 2.1179])	1.7749 (6.6804, p=0.0000, [1.4999, 2.1003])
LMSYS Users	1.5512 (4.3938, p=0.0000, [1.2753, 1.8868])	3.0860 (8.2316, p=0.0000, [2.3598, 4.0358])

3.27. Supplementary Table 27: Study 2: Ingroup solidarity and outgroup hostility of LLMs fine-tuned with partisan Twitter data.

Each value represents an odds ratio from a separate logistic regression fitted on a total of 4,000 ingroup and outgroup sentences from a Twitter (now X) Republican and Democrat-finetuned LLMs predicting whether the sentence is positive (for ingroup solidarity) based on whether the sentence is ingroup (vs. outgroup), and negative (for outgroup hostility) based on whether the sentence is outgroup (vs. ingroup), and control variables including fine-tuning party (Republican or Democrat). Tests are two-sided, and p-values are not adjusted for multiple comparisons. Base model results included for comparison. Sentence sentiment determined using RoBERTa. See Methods for more details. Data are presented as OR (z value, p value, 95% CI).

	Ingroup Solidarity	Outgroup Hostility
Partisan		
GPT-2-124M	3.8242 (18.5364, p=0.0000, [3.3185, 4.4069])	5.4413 (23.5855, p=0.0000, [4.7268, 6.2638])
GPT-2-Medium-355M	4.0140 (19.3188, p=0.0000, [3.4861, 4.6218])	5.3078 (23.2288, p=0.0000, [4.6105, 6.1105])
GPT-2-Large-774M	4.4810 (19.7047, p=0.0000, [3.8600, 5.2019])	6.0440 (24.9640, p=0.0000, [5.2479, 6.9610])
GPT-2-XL-1.5B	4.7681 (20.2998, p=0.0000, [4.1006, 5.5442])	6.8527 (26.3372, p=0.0000, [5.9383, 7.9080])
BLOOM-560M	4.7940 (19.6887, p=0.0000, [4.1015, 5.6035])	7.5103 (27.1978, p=0.0000, [6.4946, 8.6848])
BLOOM-1B1	4.1940 (19.0249, p=0.0000, [3.6181, 4.8615])	5.7127 (24.1677, p=0.0000, [4.9598, 6.5799])
BLOOM-1B7	5.6413 (21.7919, p=0.0000, [4.8284, 6.5911])	7.2871 (26.9485, p=0.0000, [6.3070, 8.4195])
BLOOM-3B	5.4591 (21.8517, p=0.0000, [4.6882, 6.3568])	7.0236 (26.5135, p=0.0000, [6.0811, 8.1123])
BLOOMZ-560M	3.7507 (17.3355, p=0.0000, [3.2300, 4.3554])	5.4494 (23.8488, p=0.0000, [4.7406, 6.2642])
BLOOMZ-1B1	4.7430 (20.0177, p=0.0000, [4.0725, 5.5239])	6.8683 (26.1092, p=0.0000, [5.9433, 7.9373])
BLOOMZ-1B7	4.9484 (20.5441, p=0.0000, [4.2483, 5.7639])	7.5742 (27.0527, p=0.0000, [6.5408, 8.7709])
BLOOMZ-3B	5.6363 (21.6443, p=0.0000, [4.8194, 6.5917])	7.9784 [illegible].8459, p=0.0000, [6.8934, 9.2342])
Base		
GPT-2-124M	2.5259 (9.5648, p=0.0000, [2.0891, 3.0541])	2.3426 (7.2767, p=0.0000, [1.8626, 2.9463])
GPT-2-Medium-355M	2.3156 (8.6639, p=0.0000, [1.9150, 2.8000])	1.9455 (5.8661, p=0.0000, [1.5576, 2.4300])
GPT-2-Large-774M	2.1572 (8.0283, p=0.0000, [1.7881, 2.6026])	2.4531 (7.6886, p=0.0000, [1.9515, 3.0836])
GPT-2-XL-1.5B	2.2033 (8.1656, p=0.0000, [1.8227, 2.6633])	1.8026 (5.1603, p=0.0000, [1.4411, 2.2547])
BLOOM-560M	1.5990 (5.1445, p=0.0000, [1.3372, 1.9121])	1.5763 (3.1016, p=0.0019, [1.1823, 2.1015])
BLOOM-1B1	1.5792 (5.0028, p=0.0000, [1.3204, 1.8888])	2.0670 (5.0556, p=0.0000, [1.5599, 2.7389])
BLOOM-1B7	2.5394 (9.7903, p=0.0000, [2.1072, 3.0602])	2.7550 (6.7809, p=0.0000, [2.0555, 3.6927])
BLOOM-3B	1.8172 (6.4118, p=0.0000, [1.5139, 2.1812])	2.0774 (5.3739, p=0.0000, [1.5912, 2.7123])
BLOOMZ-560M	1.0209 (0.2187, p=0.8269, [0.8483, 1.2286])	1.1262 (0.7787, p=0.4361, [0.8351, 1.5187])
BLOOMZ-1B1	1.8232 (6.4153, p=0.0000, [1.5175, 2.1903])	1.4238 (2.3581, p=0.0184, [1.0615, 1.9098])
BLOOMZ-1B7	1.6963 (5.2438, p=0.0000, [1.3923, 2.0668])	1.1341 (0.8923, p=0.3722, [0.8602, 1.4951])
BLOOMZ-3B	1.7285 (5.7430, p=0.0000, [1.4340, 2.0835])	1.1479 (0.9929, p=0.3208, [0.8743, 1.5072])

3.28. Supplementary Table 28: Study 2: Ingroup solidarity and outgroup hostility of LLMs fine-tuned with curated partisan Twitter data.

Each value represents an odds ratio from a separate logistic regression fitted on a total of 2,000 ingroup and outgroup sentences from a party-finetuned LLMc (Republican or Democrat Twitter (now X) data) predicting whether the sentence is positive (for ingroup solidarity) based on whether the sentence is ingroup (vs. outgroup), and negative (for outgroup hostility) based on whether the sentence is outgroup (vs. ingroup), and control variables. Tests are two-sided, and p-values are not adjusted for multiple comparisons. Sentence sentiment determined using RoBERTa. See Methods for more details. Data are presented as OR (z value, p value, 95% CI).

	Ingroup Solidarity	Outgroup Hostility
GPT-2-124M	2.5259 (9.5648, p=0.0000, [2.0891, 3.0541])	2.3426 (7.2767, p=0.0000, [1.8626, 2.9463])
Republican		
Republican GPT-2-124M	5.1485 (15.4114, p=0.0000, [4.1799, 6.3414])	7.7956 (19.3419, p=0.0000, [6.3311, 9.5990])
50% Ingroup Positive	4.9441 (14.8938, p=0.0000, [4.0063, 6.1013])	7.8256 (19.0823, p=0.0000, [6.3350, 9.6671])
50% Outgroup Negative	5.0133 (15.8051, p=0.0000, [4.1049, 6.1228])	6.7289 (17.9431, p=0.0000, [5.4639, 8.2867])
50% Both	4.4615 (14.7797, p=0.0000, [3.6589, 5.4401])	6.6575 (17.8827, p=0.0000, [5.4085, 8.1950])
0% Ingroup Positive	2.5960 (8.1965, p=0.0000, [2.0665, 3.2612])	4.0462 (14.5644, p=0.0000, [3.3524, 4.8835])
0% Outgroup Negative	3.1826 (11.8362, p=0.0000, [2.6274, 3.8552])	5.0454 (15.1714, p=0.0000, [4.0934, 6.2187])
0% Both	1.4426 (3.5309, p=0.0004, [1.1771, 1.7681])	3.1834 (11.9974, p=0.0000, [2.6347, 3.8464])
Democrat		
Democrat GPT-2-124M	2.9251 (10.7627, p=0.0000, [2.4058, 3.5565])	3.9044 (13.8563, p=0.0000, [3.2202, 4.7340])
50% Ingroup Positive	2.9726 (10.6320, p=0.0000, [2.4317, 3.6338])	3.7065 (13.6347, p=0.0000, [3.0702, 4.4745])
50% Outgroup Negative	2.5592 (9.5429, p=0.0000, [2.1100, 3.1040])	3.3648 (12.4022, p=0.0000, [2.7777, 4.0761])
50% Both	2.0919 (7.3948, p=0.0000, [1.7202, 2.5438])	2.7734 (10.6807, p=0.0000, [2.2999, 3.3443])
0% Ingroup Positive	1.4105 (3.1007, p=0.0019, [1.1349, 1.7530])	2.0005 (7.5688, p=0.0000, [1.6717, 2.3939])
0% Outgroup Negative	2.0438 (7.5726, p=0.0000, [1.6986, 2.4591])	3.0274 (10.9081, p=0.0000, [2.4810, 3.6941])
0% Both	1.1182 (1.0824, p=0.2791, [0.9134, 1.3689])	1.5682 (4.9650, p=0.0000, [1.3130, 1.8730])
