# Supplementary material for: Generative language models exhibit social identity biases
Source: Nat Comput Sci. 2024 Dec 12;5(1):65–75. doi: 10.1038/s43588-024-00741-1 (PMC11774750; doi:10.1038/s43588-024-00741-1)
Supplement: Supplementary file 2 — Reporting Summary [file 43588_2024_741_MOESM2_ESM.pdf]

Corresponding author(s): Tiancheng Hu, Yara Kyrychenko
Last updated by author(s): Nov 4, 2024

Reporting Summary

Nature Portfolio wishes to improve the reproducibility of the work that we publish. This form provides structure for consistency and transparency in reporting. For further information on Nature Portfolio policies, see our Editorial Policies and the Editorial Policy Checklist.

Statistics

For all statistical analyses, confirm that the following items are present in the figure legend, table legend, main text, or Methods section.

n/a	Confirmed	
☐	☒	The exact sample size (*n*) for each experimental group/condition, given as a discrete number and unit of measurement
☐	☒	A statement on whether measurements were taken from distinct samples or whether the same sample was measured repeatedly
☐	☒	The statistical test(s) used AND whether they are one- or two-sided<br>*Only common tests should be described solely by name; describe more complex techniques in the Methods section.*
☐	☒	A description of all covariates tested
☐	☒	A description of any assumptions or corrections, such as tests of normality and adjustment for multiple comparisons
☐	☒	A full description of the statistical parameters including central tendency (e.g. means) or other basic estimates (e.g. regression coefficient) AND variation (e.g. standard deviation) or associated estimates of uncertainty (e.g. confidence intervals)
☐	☒	For null hypothesis testing, the test statistic (e.g. *F*, *t*, *r*) with confidence intervals, effect sizes, degrees of freedom and *P* value noted<br>*Give P values as exact values whenever suitable.*
☒	☐	For Bayesian analysis, information on the choice of priors and Markov chain Monte Carlo settings
☐	☒	For hierarchical and complex designs, identification of the appropriate level for tests and full reporting of outcomes
☐	☒	Estimates of effect sizes (e.g. Cohen's *d*, Pearson's *r*), indicating how they were calculated

Our web collection on statistics for biologists contains articles on many of the points above.

Software and code

Policy information about availability of computer code

Data collection	Data consists of sentences generated by Large Language Models when prompted with "We are" or "They are".<br>Our code is based on Python 3.10. Our code and data are available at https://doi.org/10.17605/OSF.IO/9HT32.

Data analysis	All data was analyzed in R.<br>R version 4.3.1 (2023-06-16)<br>Platform: aarch64-apple-darwin20 (64-bit)<br>Running under: macOS Monterey 12.2.1<br><br>attached base packages:<br>stats graphics grDevices utils datasets methods base<br><br>other attached packages:<br>stminsights_0.4.2 stm_1.3.6.1 stmCorrViz_1.3<br>qdapRegex_0.7.8 igraph_1.5.1 quanteda.textstats_0.96.4<br>quanteda_3.3.1 patchwork_1.2.0 sjPlot_2.8.15<br>ggraph_2.1.0 cowplot_1.1.1 gridExtra_2.3<br>stargazer_5.2.3 metafor_4.6-0 numDeriv_2016.8-1.1<br>metadat_1.2-0 lme4_1.1-35.1 Matrix_1.6-3<br>scales_1.3.0 dplyr_1.1.4 dotwhisker_0.7.4<br>ggplot2_3.5.1 jtools_2.2.2

For manuscripts utilizing custom algorithms or software that are central to the research but not yet described in published literature, software must be made available to editors and reviewers. We strongly encourage code deposition in a community repository (e.g. GitHub). See the Nature Portfolio guidelines for submitting code & software for further information.

Data

Policy information about availability of data

All manuscripts must include a data availability statement. This statement should provide the following information, where applicable:

- Accession codes, unique identifiers, or web links for publicly available datasets
- A description of any restrictions on data availability
- For clinical datasets or third party data, please ensure that the statement adheres to our policy

Our code and data are available at https://doi.org/10.17605/OSF.IO/9HT32.

Human research participants

Policy information about studies involving human research participants and Sex and Gender in Research.

Reporting on sex and gender	N/A
Population characteristics	N/A
Recruitment	N/A
Ethics oversight	N/A

Note that full information on the approval of the study protocol must also be provided in the manuscript.

Field-specific reporting

Please select the one below that is the best fit for your research. If you are not sure, read the appropriate sections before making your selection.

☐ Life sciences ☒ Behavioural & social sciences ☐ Ecological, evolutionary & environmental sciences

For a reference copy of the document with all sections, see nature.com/documents/nr-reporting-summary-flat.pdf

Behavioural & social sciences study design

All studies must disclose on these points even when the disclosure is negative.

Study description	In this quantitative study, we collect LLM-generated sentences starting with "We are" or "They are" and evaluate their sentiment (positive, neutral, negative) to calculate the levels of ingroup solidarity and outgroup hostility per LLM.
Research sample	Large Language Models (LLMs)
Sampling strategy	We collect randomly-generated sentences. No sample-size calculations were performed.
Data collection	We prompt LLMs to generate sentences starting with "We are" or "They are".

Data collection	In study 1, we obtain data from several major LLM pretraining corpora, including C4, OpenWebText, an open source replica of GPT-2 training corpus, OLM (November/December 2022 Common Crawl data), and The Pile.<br><br>In study 2, we obtain a dataset of US Republican and Democrat posts from Twitter (now X) from https://aclanthology.org/2022.coling-1.593/.<br><br>In study 3, we obtained sentences from human-LLM conversations starting with "We are" or "They are" from publicly available datasets (WildChat and LMSYS; https://arxiv.org/abs/2405.01470 and https://arxiv.org/abs/2309.11998).
Timing	May 2023 - March 2024
Data exclusions	N/A
Non-participation	N/A, as our participants are LLMs
Randomization	N/A, as our participants are LLMs

Reporting for specific materials, systems and methods

We require information from authors about some types of materials, experimental systems and methods used in many studies. Here, indicate whether each material, system or method listed is relevant to your study. If you are not sure if a list item applies to your research, read the appropriate section before selecting a response.

Materials & experimental systems

n/a	Involved in the study
☒	☐ Antibodies
☒	☐ Eukaryotic cell lines
☒	☐ Palaeontology and archaeology
☒	☐ Animals and other organisms
☒	☐ Clinical data
☒	☐ Dual use research of concern

Methods

n/a	Involved in the study
☒	☐ ChIP-seq
☒	☐ Flow cytometry
☒	☐ MRI-based neuroimaging
